# Monitoring of compound resting membrane potentials of cell cultures with ratiometric genetically encoded voltage indicators

**DOI:** 10.1038/s42003-021-02675-0

**Published:** 2021-10-07

**Authors:** Philipp Rühl, Johanna M. Langner, Jasmin Reidel, Roland Schönherr, Toshinori Hoshi, Stefan H. Heinemann

**Affiliations:** 1 Center for Molecular Biomedicine, Department of Biophysics, Friedrich Schiller University Jena and Jena University Hospital, D-07745 Jena, Germany; 2 Department of Physiology, University of Pennsylvania, Philadelphia, PA USA

**Keywords:** Ion transport, Mechanisms of disease

## Abstract

The cellular resting membrane potential (*V*_m_) not only determines electrical responsiveness of excitable cells but also plays pivotal roles in non-excitable cells, mediating membrane transport, cell-cycle progression, and tumorigenesis. Studying these processes requires estimation of *V*_m_, ideally over long periods of time. Here, we introduce two ratiometric genetically encoded *V*_*m*_ indicators, rArc and rASAP, and imaging and analysis procedures for measuring differences in average resting *V*_m_ between cell groups. We investigated the influence of ectopic expression of K^+^ channels and their disease-causing mutations involved in Andersen-Tawil (Kir2.1) and Temple-Baraitser (K_V_10.1) syndrome on median resting *V*_m_ of HEK293T cells. Real-time long-term monitoring of *V*_m_ changes allowed to estimate a 40–50 min latency from induction of transcription to functional Kir2.1 channels in HEK293T cells. The presented methodology is readily implemented with standard fluorescence microscopes and offers deeper insights into the role of the resting *V*_*m*_ in health and disease.

## Abstract (web summary)

Rühl et al. report the generation of ratiometric genetically encoded voltage indicators (GEVIs) and establish that they can be used in high-throughput automated imaging to measure compound membrane potential (Vm) in mammalian cells. This method is implementable with standard fluorescence microscopes and has the potential to offer insights into the role of the resting Vm in health and disease.

## Introduction

The cell membrane potential (*V*_m_) and its changes are key to cellular excitability and many other physiological/pathophysiological processes, such as membrane transport, cell-cycle progression, embryonic development, and cancer, contributing to the overall driving force on other ions and solutes^1–8^. Knowing resting *V*_m_ is therefore essential for understanding the underlying mechanisms.

Resting *V*_m_ is typically measured with electrophysiological methods, such as sharp microelectrodes or whole-cell patch clamp. However, the necessity to approach single cells with one electrode at a time renders these methods tedious and time consuming. Furthermore, disturbing the cell’s integrity by rupturing the membrane and replacing the intracellular medium with a predefined pipette solution, as in the whole-cell patch-clamp method, severely limit the applicability of electrode-based methods for comparing *V*_m_ of different populations of cells. Much less invasive are voltage-sensitive organic dyes such as Di-4-ANEPPS, or FRET-oxonol systems, which have been used to study changes in *V*_m_ for decades^9–11^. However, these organic dyes have notable drawbacks: limitations in the targeting to specific cell populations, the need of carefully optimized loading procedures, potential cytotoxicity, or direct interference with ion transport proteins^11,12^. Some of these limitations are minimized in genetically encoded voltage indicators (GEVIs).

The potentiometric GEVIs ArcLight, ASAP, and more recently developed derivates, based on voltage-sensitive phosphatases of, e.g., *Ciona intestinalis* or *Gallus gallus*, feature bright fluorescence and a large dependence of fluorescence intensity on *V*_m_ changes^13–16^. Archaerhodopsin-based sensors are fast and have a large dynamic range but typically suffer from low quantum yields^17,18^. Newly developed GEVIs are usually optimized for the detection of fast changes in *V*_m_, such as neuronal action potentials^19^. Only a small fraction of research has focused on calibrating the signals with respect to the absolute *V*_m_^20,21^. However, changes of *V*_m_ on time scales of minutes to days elicited by altered activity and/or expression of ion channels, electrogenic transporters, and pumps are proposed to occur during cell-cycle and tumor progression^1^. The investigation of such processes therefore requires GEVIs that report resting *V*_m_ differences between cell populations or changes over extended periods of time.

For monitoring *V*_m_, existing techniques suffer from various technical limitations and application hurdles. Determination of the absolute *V*_m_ with the nonequilibrium dynamics of a rhodopsin photocycle requires a time-resolved, multiwavelength pump-probe protocol^21^. The voltage-dependent signals arising from two GEVIs, ASAP1 and CAESR, were analyzed with fluorescence lifetime imaging microscopy (FLIM) to estimate absolute *V*_m_^20^. However, FLIM needs a high-speed dedicated and specialized setup that may not be readily available in many physiology and biology laboratories, thus limiting the wide-spread adoption. Although measurement of resting *V*_m_ with ratiometric GEVIs was suggested earlier^22^, to our knowledge it has not been attempted with any of the currently available ratiometric GEVIs such as VSFP2.42 or Mermaid2^17,23–26^.

Here, we exploited the large dynamic range of the potentiometric GEVI families ArcLight and ASAP, and developed *ratiometric* variants based on ArcLight-Q239 (rArc) and ASAP2s (rASAP). Although none of the developed indicators offered single-cell *V*_m_ accuracy, we successfully deployed them in microscopy-based procedures for automated extraction of fluorescence information from high numbers of cells (>1000) to compare compound resting *V*_m_ of independent cell groups. The easy-to-implement experimental strategy of standard fluorescence microscopic image acquisition and analysis, as outlined below, enabled us to evaluate the impact of the expression of disease-relevant ion channels and channel mutants on resting *V*_m_ of large populations of cells in culture. Furthermore, long-term live-cell imaging allowed to estimate the biogenesis speed of Kir2.1 channels from triggered transactivation to functional channels in real time.

## Results

### Comparison of ratiometric GEVIs based on ArcLight and ASAP

**Fig. 1:**
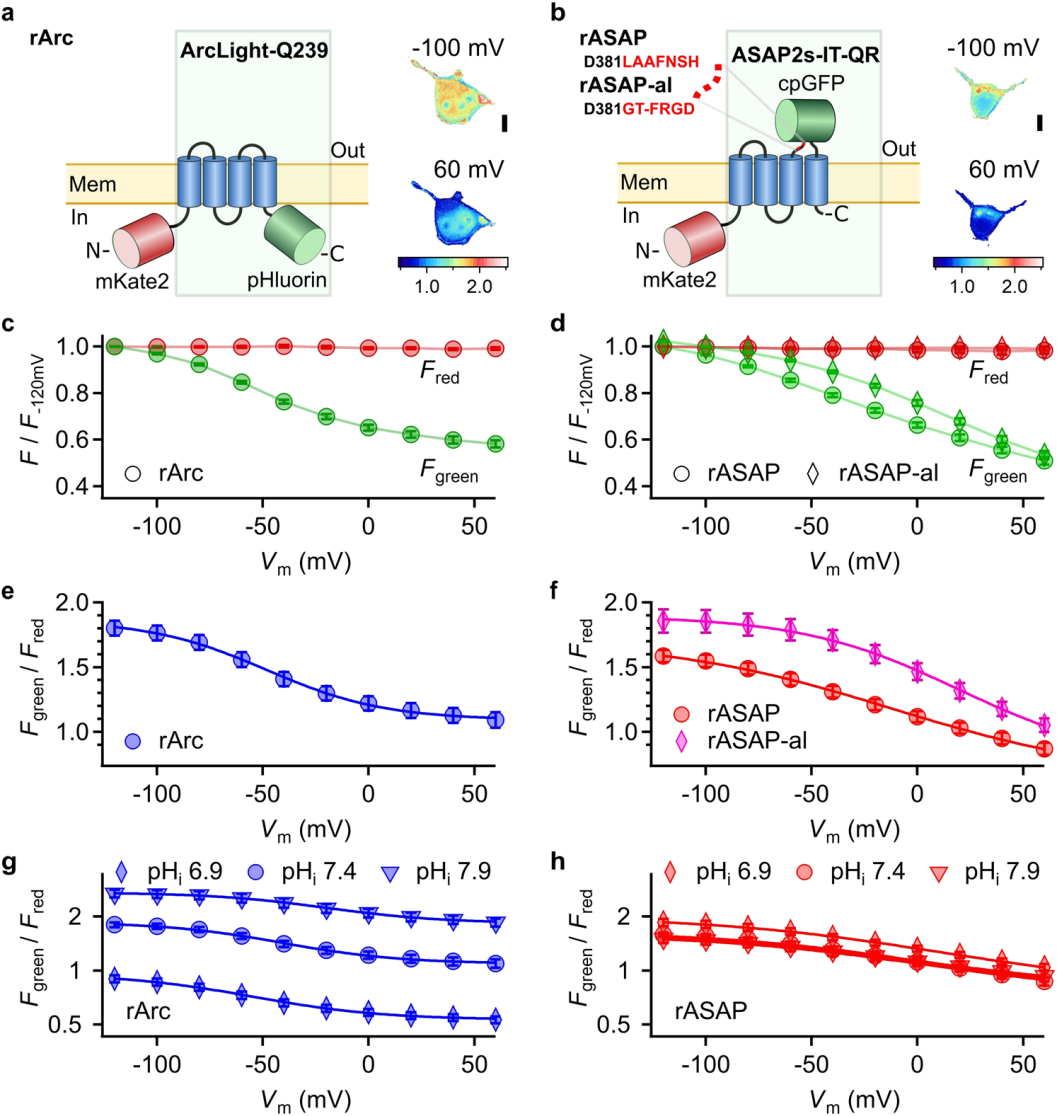
Evaluation of ratiometric GEVIs based on ArcLight and ASAP. **a**, **b** Schematics of ratiometric rArcLight-Q239 (rArc) and ratiometric ASAP2s-I67T-Q397R (rASAP). rASAP and rASAP-al differ in the linker between the S3 transmembrane segment of the voltage-sensitive phosphatase and cpGFP as indicated. Pixel-by-pixel fluorescence ratio (*F*_green_/*F*_red_) images of HEK293T cells expressing rArc (**a**) or rASAP (**b**) voltage-clamped to the indicated *V*_m_. Images were acquired with 470 nm (green) and 530 nm (red) excitation light (Supplementary Fig. 1). Calibration bar indicates *F*_green_/*F*_red_ values. Scale bar, 10 µm. **c**, **d** Normalized *F*_green_ and *F*_red_ signals of voltage-clamped cells with rArc, rASAP, or rASAP-al; *n* = 10–11. Straight lines connect data points for clarity. **e**, **f** *F*_green_/*F*_red_ from fluorescence data shown in (**c**) and (**d**) as a function of *V*_m_. The *F*_green_/*F*_red_ signal was corrected for bleaching (Supplementary Fig. 1). Regions of interests (ROIs) for data extraction included the membrane areas only. **g**, **h** *F*_green_/*F*_red_ as a function of voltage of cells expressing rArc or rASAP with intracellular solution adjusted to the indicated intracellular pH (pH_i_). Data at pH_i_ 7.4 are identical to data in (**e**) and (**f**), respectively; *n* = 7–11. Data are means ± sem. Smooth curves in **e**–**h** are the results of fits according to Eq. 1; for results see Supplementary Table 1.

We generated ratiometric GEVIs by fusing the red fluorescent protein mKate2 to the N terminus of the potentiometric GEVI ArcLight-Q239 (Fig. 1a, here termed rArc) or to ASAP2s with point mutations I67T and Q397R (Fig. 1b, here termed rASAP)^13,14,27,28^. In addition, we constructed a rASAP variant in which the linker between the transmembrane segment S3 of the voltage-sensitive phosphatase and cpGFP (Fig. 1b) was altered (rASAP-al) according to the mutations in ASAP3^16^.

The three GEVIs, rArc, rASAP and rASAP-al, produced robust fluorescence signals in HEK293T cells. The red fluorescence signals (*F*_red_) of all three GEVIs were independent of *V*_m_ when controlled by whole-cell patch clamp (Fig. 1c, d). The ratio of green to red fluorescence (*F*_green_/*F*_red_) of rASAP was smaller than that of rArc or rASAP-al at the same *V*_m_ (Fig. 1e, f) owing to relatively dimmer *F*_green_. For all GEVIs, *F*_green_ underwent rapid and saturable loss after the start of illumination caused by reversible photoswitching (Supplementary Fig. 1a, b), as reported previously for other ASAP variants^29^. In contrast to *F*_green_, *F*_red_ originating from mKate2 was stable during illumination (Supplementary Fig. 1c). To acquire images after the *F*_green_ signal stabilized, simultaneous patch-clamp and imaging experiments were performed according to the recording protocol shown in Supplementary Fig. 1d. Furthermore, residual photobleaching was compensated (Supplementary Fig. 1e). The resulting *F*_green_/*F*_red_(*V*_m_) followed a Boltzmann-type function (Eq. 1) with maximum change, midpoint voltage, and slope factor (Δ*r*, *V*_half,_ *k*_s_) of 41%, −49 mV, 27.8 mV for rArc, 63%, ­11.4 mV, −53.8 mV for rASAP, and 60%, −19 mV, 36.7 mV for rASAP-al (Fig. 1e, f and Supplementary Table 1). We excluded rASAP-al from further investigation because its sensitivity optimum (*V*_half_ ± *k*_s_) was in a voltage range unsuitable for faithful determination of average resting *V*_m_, which is typically between 0 and −100 mV^1^.

Voltage-step-induced *F*_green_ changes of rArc and rASAP followed a double-exponential time course with fast and slow components (Supplementary Fig. 2a). For a voltage step from −140 to 60 mV, a fast component time constant of 7.2 ± 0.3 ms with a relative amplitude (*a*_fast_) of 70.2 ± 1.4% and a slow component time constant of 73 ± 5 ms was determined for rASAP. For rArc, both, the fast (18.7 ± 1.1 ms, 65.4 ± 1.4%) and slow components (158 ± 14 ms) were substantially slower than for rASAP (Supplementary Fig. 2b, c).

We also designed ArcLight-Q239 fusion constructs based on the red fluorescent protein mCherry (rArc-mCherry). However, *F*_red_ images showed bright intracellular punctuate structures (Supplementary Fig. 3a), as reported previously for the FRET-based GEVIs Mermaid1 and Mermaid2β^26^. Similar punctuate structures are found with some of the DsRed-derived fluorescence proteins and were reported to originate from insufficient lysosomal fluorescence protein degradation^30^. Variant rArc-mCherry-M71T, which shifts the pK_a_ of mCherry from less than 4 to 7.8^31^, mitigated the formation of bright *F*_red_ spots (Supplementary Fig. 3a) probably because of the acidic endosomal pH. However, the mutation also decreased the intensity of the red signal by about sevenfold and substantially increased the cell-to-cell variation in the *F*_green_/*F*_red_ signal compared to rArc-mCherry and rArc (Supplementary Fig. 3b–d). While bright intracellular structures, which are likely caused by GEVI located in ER and Golgi, were also visible for all other GEVIs, we did not observe these punctuate red fluorescent structures for mKate2-based rArc (Supplementary Fig. 3a).

In live-cell imaging experiments the extracellular pH is determined by the choice of the bath solution, while the intracellular pH (pH_i_) is not directly controlled. Since ArcLight-Q239 is based on the fluorescent protein pHluorin whose strong pH sensitivity is critical for the high Δ*r* value of ArcLight-Q239^31,32^, we investigated the impact of pH_i_ variations on *F*_green_/*F*_red_(*V*_m_) of rArc and rASAP. *F*_green_/*F*_red_(*V*_m_) of rArc was particularly sensitive to pH_i_ changes: at −100 mV, *F*_green_/*F*_red_ at pH_i_ 7.9 was threefold larger than at pH_i_ 6.9 (Fig. 1g and Supplementary Table 1); this difference was greater than any measured *F*_green_/*F*_red_ change caused by manipulation of *V*_m_ at a constant pH_i_. In contrast, alteration of pH_i_ had only a minor impact on *F*_green_/*F*_red_(*V*_m_) of rASAP (Fig. 1h).

### Live-cell high-content assay for detecting changes in compound resting *V*_m_

**Fig. 2:**
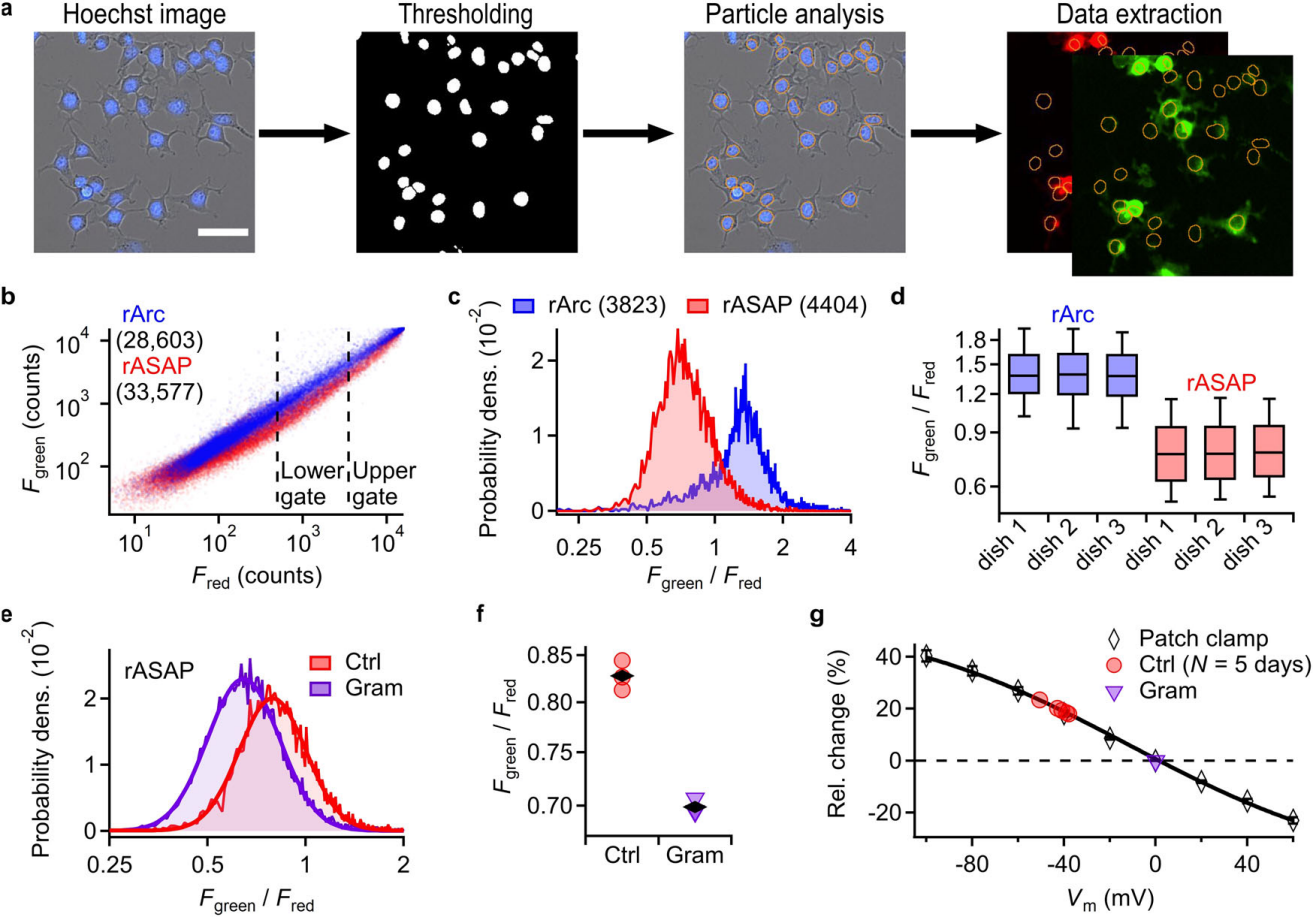
Automated high-content assay. **a** Workflow for automated extraction of fluorescence information from images of plated HEK293T cells expressing ratiometric GEVIs; cells were pretreated with the DNA-binding dye Hoechst 33342 enabling automated ROI (orange circles) generation by setting a fluorescent threshold and applying a particle analysis algorithm on the *F*_blue_ channel. *F*_blue_ and transmission light images are superimposed. Scale bar, 50 µm. For details see methods. **b** *F*_green_ as a function of *F*_red_ of >28,600 individual cells from one cell-culture dish transfected with rArc or rASAP expression vectors, extracted with the method described in **a**. Dashed lines indicate lower and upper gates used for subsequent data analysis. **c** Probability density of fluorescence ratio (*F*_green_/*F*_red_) of gated data from (**b**). **d** Boxplots of *F*_green_/*F*_red_ values from three separate culture dishes of HEK293T cells expressing rArc or rASAP. **e** *F*_green_/*F*_red_ distribution of cells from independent cell-culture dishes expressing rASAP with (purple) or without (red) the ionophore gramicidin (1 μM). The log(*F*_green_/*F*_red_) distributions were fit with Gaussian functions (solid curves, Eq. 4). **f** Median *F*_green_/*F*_red_ values from three separate cell-culture dishes (as in (**e**)). Black rhombi are mean values of the median *F*_green_/*F*_red_ values from individual dishes. **g** Normalized *F*_green_/*F*_red_(*V*_m_) of rASAP with superimposed Boltzmann-type functions (Eq. 1) (from Fig. 1f); parameters of the fit were *R*_max_ = 0.56, Δ_r_ = −178.6%, *V*_half_ = −11.5 mV, *k*_s_ = 54.0 mV. Average *V*_m_ of HEK293T cells (red) was estimated by normalization of *F*_green_/*F*_red_ of untreated cells to *F*_green_/*F*_red_ of gramicidin-treated cells, assuming full depolarization to 0 mV by gramicidin. Each data point is the average of 3 individual measurements (as in (**e**, **f**)).

One of the greatest advantages of optical *V*_m_ monitoring methods over those using electrodes is the possibility to record from thousands of cells simultaneously. We developed a high-content, microscopy-based measurement technique that allowed us to automatically extract the *F*_green_ and *F*_red_ signals of cells. HEK293T cells were treated with the blue fluorescent membrane-permeable DNA dye Hoechst 33342^33^, which highlights the cell nucleus and enables automatic generation of regions of interest (ROIs) using a thresholding procedure and a subsequent particle analysis algorithm (Fig. 2a). This method of ROI selection not only allows for automation but also minimizes the contribution of GEVI fluorescence from intracellular organelles because the cytosolic space above and below the nucleus is quite limited (Fig. 2a). In this way we acquired and automatically analyzed *F*_green_/*F*_red_ data from 10,000–60,000 cells per culture dish (Fig. 2b). Non-transfected and low-expressing cells as well as cells with very large fluorescence were eliminated by setting cutoffs based on *F*_red_ (Fig. 2b). Although both rArc and rASAP showed a relatively large cell-to-cell variation in *F*_green_/*F*_red_ (Fig. 2c, d), the median of the *F*_green_/*F*_red_ distribution proved to be a robust data descriptor: for example, for only 1000 cells the estimated standard deviation of the median, based on resampling of the cell-to-cell distribution of *F*_green_/*F*_red,_ was 1% for rASAP (Fig. 2e), i.e. smaller than the symbol size in Fig. 2f. To estimate the dish-to-dish variation, we measured cells expressing rASAP on five independent days with three dishes each and achieved an average standard deviation of the median *F*_green_/*F*_red_ from dish to dish of 1.8 ± 0.3%.

To derive a rough estimate of the average *V*_m_ of the cell population based on the medians of the *F*_green_/*F*_red_ signals of rASAP on a microscope without patch-clamp control, we used gramicidin, an ionophore that depolarizes cells to about 0 mV^9^. We normalized the average median *F*_green_/*F*_red_ values of three independent cell-culture dishes of untreated cells to the average *F*_green_/*F*_red_ value of three cell-culture dishes with gramicidin-treated cells on the same day (Fig. 2e–g) and used the normalized *F*_green_/*F*_red_(*V*_m_) from patch-clamp experiments as calibration curve (Fig. 1f). Samples with gramicidin exhibited a lower *F*_green_/*F*_red_ median value than samples without gramicidin. We repeated the measurements on five different days and found a consistent shift in the median *F*_green_/*F*_red_ value of samples treated with gramicidin by −16.5 ± 0.7% (standard deviation of 1.4%) compared to untreated cells, indicating a stable average *V*_m_ value of the HEK293T cell populations.

Using the parameters from the patch-clamp calibration curve and the *F*_green_/*F*_red_ of gramicidin-treated cells as reference for 0 mV, we estimated an average resting *V*_m_ of about −40 mV for HEK293T cell populations, which is in good agreement with previous reports^10,34^. Notably, the width (geometric standard deviation) of the *F*_green_/*F*_red_ distribution of gramicidin-treated cells (factor 1.36 ± 0.03, sd) was not diminished but rather slightly increased compared to untreated cells (1.31 ± 0.01, sd, Fig. 2e), suggesting that the cell-to-cell variation in *F*_green_/*F*_red_ is mainly related to voltage-independent variations in the recorded *F*_green_/*F*_red_ signal and thus precluding inferences about *V*_m_ of individual cells. Nevertheless, the stability and reproducibility render the median values of *F*_green_/*F*_red_ distributions as robust descriptors of *V*_m_ of large cell populations.

We also performed similar experiments with cancer cell lines derived from mouse neuroblastoma (Neuro2A) and human melanoma (A375); in both cell lines, robust expression of rASAP was observed and cell depolarization by application of gramicidin yielded shifts in the medians of the *F*_green_/*F*_red_ distributions by 16% and 23% for Neuro2A and A375 cells, respectively (Supplementary Fig. 4).

**Fig. 3:**
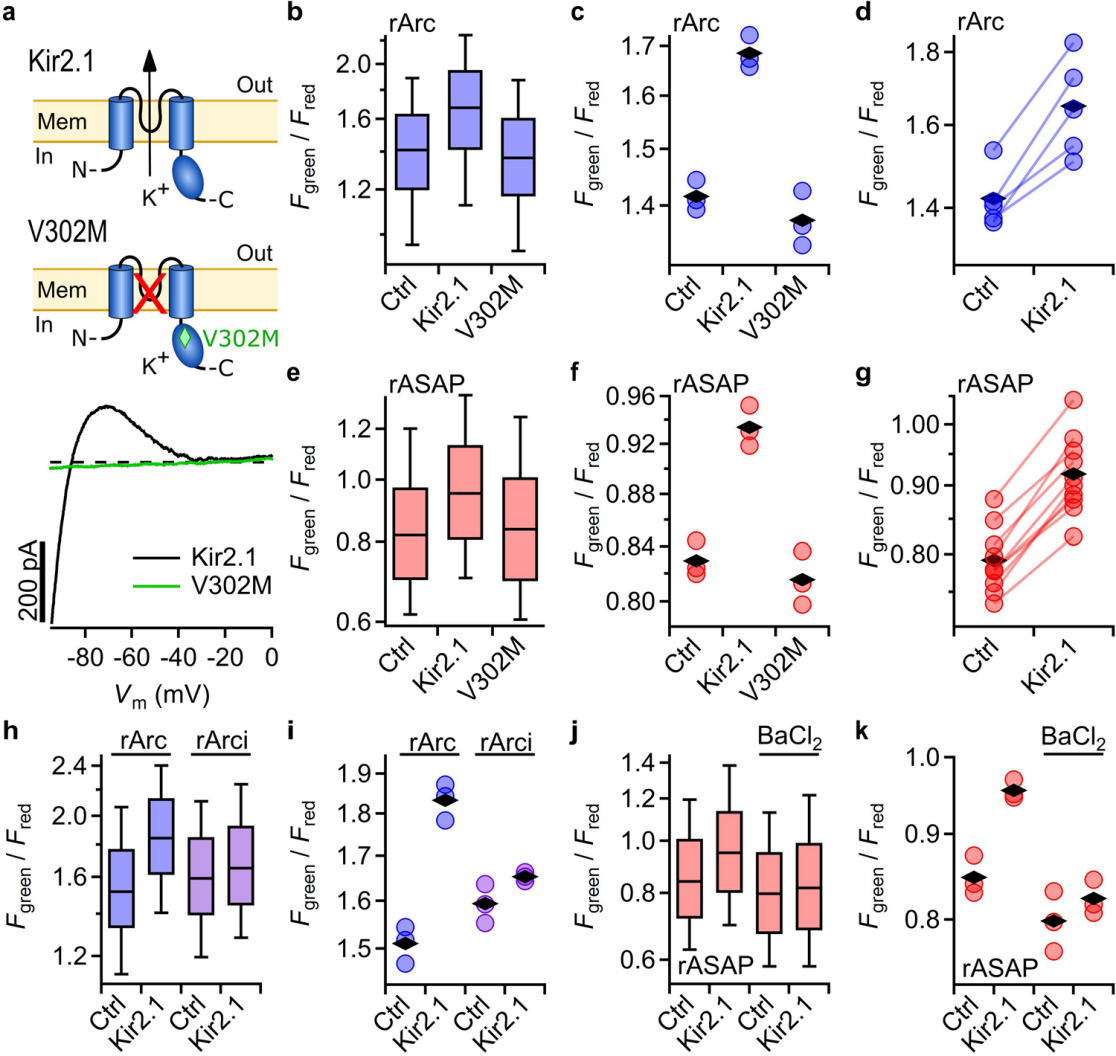
Measurement of Kir2.1-induced HEK293T cell hyperpolarization. **a** Schematics of one of four subunits forming a functional inward rectifier K^+^ channel Kir2.1 or the nonconducting variant V302M, which causes the Andersen-Tawil syndrome; bottom, representative current traces as function voltage for mouse Kir2.1 (black) or variant V302M (green), expressed in HEK293T and recorded in whole-cell patch-clamp configuration. Dashed line indicates zero current level. **b** Boxplots of *F*_green_/*F*_red_ from images of HEK293T cells expressing rArc alone (Ctrl) or coexpressing Kir2.1 or Kir2.1-V302M. **c** Median ratio values of three independent samples measured on the same day; black rhombi indicate means of the median values. **d** *F*_green_/*F*_red_ from samples with rArc (Ctrl) or rArc-Kir2.1 from five different measurement days. Each data point is the mean of at least three individual median measurements (as in (**b**, **c**)). Data points connected by lines were acquired on the same day. **e**–**g** As in (**b**–**d**) but with cells coexpressing rASAP; from 10 days in (**g**). **h** Boxplots of *F*_green_/*F*_red_ from cells expressing rArc or rArci, a rArc variant with reduced voltage sensitivity (Supplementary Fig. 6), with and without Kir2.1 and (**i**) median values of three separate samples. **j** Boxplots of *F*_green_/*F*_red_ of cells expressing rASAP alone or coexpressing mKir2.1 with or without the mKir2.1 blocker BaCl_2_ (1 mM) and **k**, median ratio values of three independent samples.

To examine if rArc and rASAP are also suited to detect physiological changes in compound resting *V*_m_, e.g., induced by the activity of ion channels, we overexpressed the mouse inward rectifier K^+^ channel Kir2.1 (mKir2.1), which stabilizes a negative *V*_m_ in excitable cells^35^. Previous reports showed that Kir2.1 is also capable of hyperpolarizing HEK293T cells in a physiological saline solution^16^. To exclude the possibility of a nonspecific interference of the channel proteins with the GEVIs, we also expressed variant Kir2.1-V302M. This mutation causes the Andersen-Tawil syndrome in humans and abolishes the ion conduction without influencing the channel’s plasma membrane localization^35^. Whole-cell patch-clamp recordings confirmed the strong inwardly rectifying K^+^ current of the wild-type channel and lack of current for the Andersen-Tawil mutant (Fig. 3a).

For rArc and rASAP, *F*_green_ alone failed to show systematic changes in response to mKir2.1 expression (Supplementary Fig. 5). In contrast, the ratio of *F*_green_ and *F*_red_ of cells expressing mKir2.1 was elevated in every experiment on every measurement day for rArc (Fig. 3b–d) as well as for rASAP (Fig. 3e–g), justifying the ratiometric approach in rArc and rASAP to compensate for variations in GEVI expression intensity. We observed an increase of the median *F*_green_/*F*_red_ by Kir2.1 overexpression of 16.1 ± 2.6% (from 5 days) for rArc and 15.9 ± 1.2% (from 10 days) for rASAP. Cells expressing the non-conducting variant Kir2.1-V302M yielded *F*_green_/*F*_red_ values similar to those in control cells for both sensor types.

Since *F*_green_/*F*_red_ of rArc strongly depended on pH_i_, we also generated rArci, a rArc variant with charge neutralizing mutations in the voltage-sensing domain, which were shown to be involved in voltage-sensor movement^36^. As expected, rArci had a markedly decreased voltage sensitivity but an almost unaltered pH_i_ sensitivity of the *F*_green_/*F*_red_ signal (Supplementary Fig. 6) and, thus, served as a pH_i_ control. The increase in *F*_green_/*F*_red_ of rArc in cells overexpressing Kir2.1 was strongly diminished in rArci (Fig. 3h, i), indicating that the observed signal change in rArc due to Kir2.1 overexpression was mainly caused by a change in *V*_m_ and not in pH_i_. To independently validate that it is the ion conduction through Kir2.1 that leads to hyperpolarization, we treated cells with the Kir2.1 blocker Ba^2+^. BaCl_2_ decreased rASAP *F*_green_/*F*_red_ to levels similar to those found in cells without Kir2.1 (Fig. 3j, k), demonstrating that the observed hyperpolarization is caused by Kir2.1 channel activity.

In conclusion, both rArc and rASAP, in combination with the assays described, are capable of resolving differences in resting *V*_m_ of different cell groups induced by physiological stimuli. Because of the higher dynamic range and pH_i_ stability, we performed further experiments exclusively with rASAP.

### Impact of voltage-gated K^+^ channels on cellular resting *V*_m_

Various cancer cell types possess markedly upregulated levels of K^+^ channels which can promote hyperpolarization^1^. For example, the voltage-gated K^+^ channel K_V_10.1 (ether à go-go 1, EAG1, *KCNH*1) was found overexpressed in 80% of biopsies from the most common solid tumors^4^. The large-conductance voltage- and Ca^2+^-activated K^+^ channel Slo1 (BK, *KCNMA*1) was found upregulated in prostate cancer cells^2^. Furthermore, gain-of-function mutations of K_V_10.1 are associated with severe genetic diseases, such as the autosomal dominant Temple-Baraitser syndrome and other developmental disorders causing dysmorphic physical features^6^. The mechanisms underlying these pathophysiological processes may be related to changes in resting *V*_m_ of cells expressing these channels. Because channel open probabilities of Slo1 and K_V_10.1 are strongly affected by intracellular factors such as Ca^2+^, Mg^2+^, or pH_i_^37–39^, the channels’ impact on cellular *V*_m_ under resting conditions is hardly accessible with standard electrophysiological methods.

**Fig. 4:**
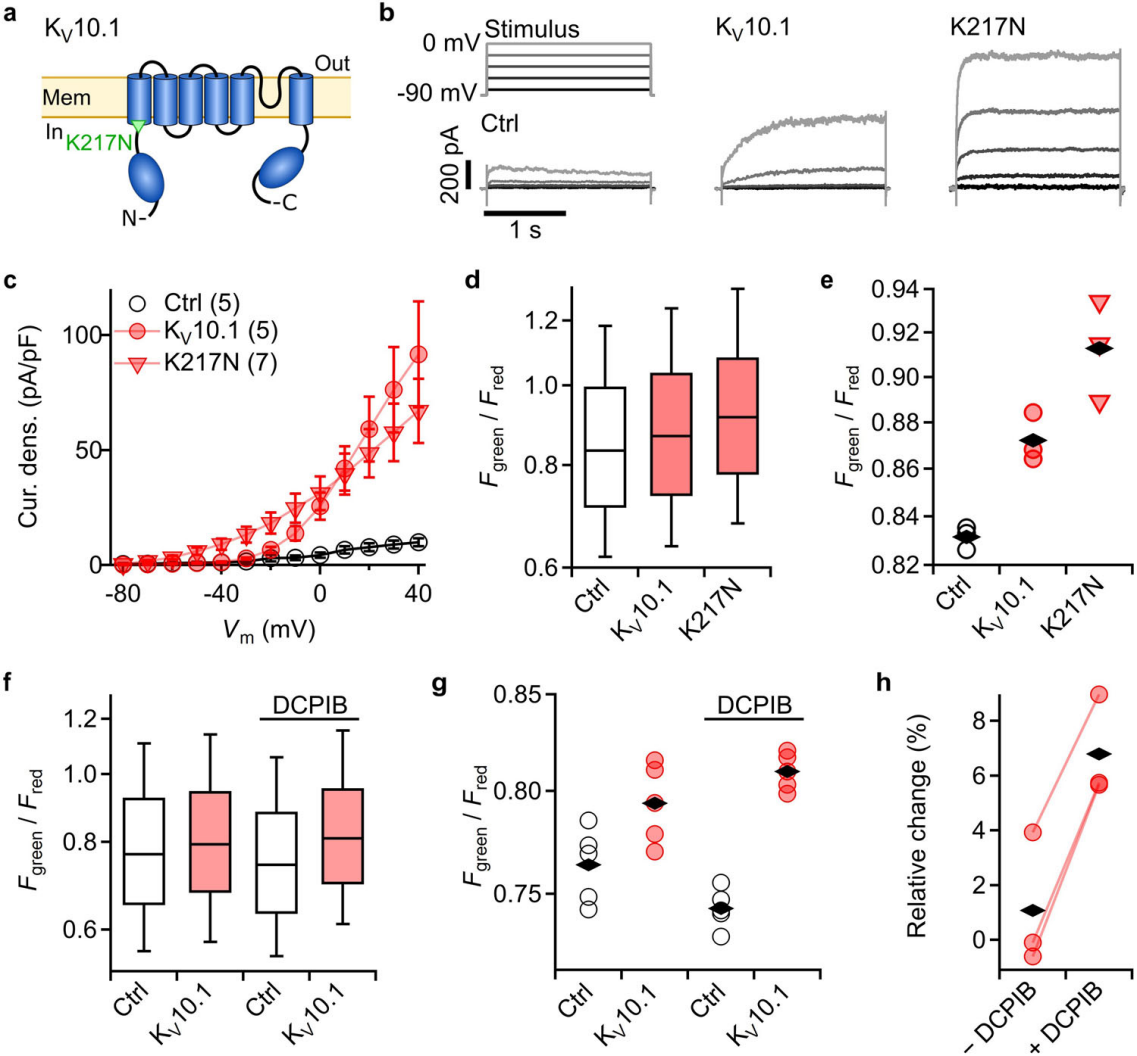
Kv10.1-K217N, a causative mutation for Temple-Baraitser syndrome, strongly hyperpolarizes HEK293T cells. **a** Schematic of one of four subunits forming a functional human ether à go-go channel (Kv10.1). Point mutation K217N shifts the current-voltage relationship to more negative voltages. **b** Representative current traces of HEK293T cells expressing rASAP alone (Ctrl) or coexpressing Kv10.1 or Kv10.1-K217N. Current traces were acquired using the depicted voltage pulse protocol. Control recordings were acquired from cells without Kv10.1. **c** Current density as a function of the voltage of cells expressing rASAP alone or coexpressing the indicated Kv10.1 variant. Data points are means ± sem, *n* is given in parentheses. Straight lines connect data points for clarity. **d** Boxplots of *F*_green_/*F*_red_ from HEK293T cells expressing rASAP alone or coexpressing the indicated variant. **e** Median ratio values of three independent samples (as in (**d**)) measured on the same day; black rhombi are average values; cells >4400 for each measurement. **f** *F*_green_/*F*_red_ of cells expressing K_V_10.1 with and without 10 µM of the Cl^−^ channel blocker DCPIB. **g** Median ratio values of five independent samples (as in (**f**)). **h** Relative change in *F*_green_/*F*_red_ of cells with K_V_10.1 over control cells with and without DCPIB from three days; connected data points are average changes acquired on the same day.

We investigated the impact of Kv10.1 channels and the Temple-Baraitser mutation Kv10.1-K217N on *V*_m_ in HEK293T cells (Fig. 4). While a gain-of-function phenotype of this mutation has been described^6^, the impact on *V*_m_ has not been experimentally addressed. Robust functional expression of wild-type and Kv10.1-K217N channels was electrophysiologically confirmed (Fig. 4b, c). At negative voltages, cells expressing the variant K217N exhibited greater outward currents than those with wild-type channels (Fig. 4c), suggesting that the K217N variant is capable of hyperpolarizing the cells. The cells expressing wild-type Kv10.1 had a modestly greater *F*_green_/*F*_red_ of 3.7 ± 1.9% (from 3 days) compared with cells without ectopic channel expression (Fig. 4d, e). Cells expressing the Temple-Baraitser mutant K217N showed a greater change in the fluorescence ratio signal of 12.7 ± 2.2% (from 3 days) over control cells (Fig. 4d, e). This greater hyperpolarization by Kv10.1-K217N is in good agreement with the electrophysiological results obtained in individual cells (Fig. 4b, c).

The impact of Kv10.1-K217N on *V*_m_ of HEK293T cells was pronounced and readily measured with this approach. The increase in *F*_green_/*F*_red_ of wild-type K_V_10.1 expressing cells compared with cells expressing only rASAP, however, was small and not seen on every day (3 out of 5 days). A potential confounding factor could be the selection of the host cells. For example, the observed day-to-day variation in K_V_10.1-induced hyperpolarization is expected because HEK293T cells are electrically coupled^10^ and therefore may depend on variations in cell density and transfection efficiency. Endogenous Cl^−^  channels, such as VRAC Cl^−^ channels that are expressed in HEK293T cells^40^, may furthermore counteract cell hyperpolarization by K_V_10.1. This notion is supported by experiments with the Cl^−^channel blocker DCPIB. DCPIB lowered *F*_green_/*F*_red_ compared to the control cells, while it increased *F*_green_/*F*_red_ in cells expressing K_V_10.1 (Fig. 4f, g). Independent measurements on 3 different days revealed that application of 10 µM DCPIB increased the average change in *F*_green_/*F*_red_ of K_V_10.1-expressing cells over control cells to 6.8 ± 1.1% (Fig. 4h). DCPIB thus eliminated part of the shunting effect of background Cl^−^ conductance and enhanced the resolution of the impact of Kv10.1 expression.

Overexpression of pore-forming human Slo1 (hSlo1) subunits of large-conductance Ca^2+^- and voltage-dependent BK channels did not change rASAP’s *F*_green_/*F*_red_ signal, consistent with these channels being mostly closed near resting *V*_m_. However, hSlo1 variants with the voltage dependence of opening shifted in the negative direction did increase *F*_green_/*F*_red_ commensurate with their electrophysiological properties (Supplementary Fig. 7).

### Monitoring of functional ion channel biogenesis under live-cell culture conditions

Processes that involve altered expression of ion channels, transporters, and pumps can occur on timescales of minutes to several hours. Detecting the impact on *V*_m_ of such slow events requires a sensor that is stable under cell-culture conditions for the respective durations. Ratiometric GEVIs may be suited for this purpose because the ratioing should compensate variations in GEVI expression levels.

**Fig. 5:**
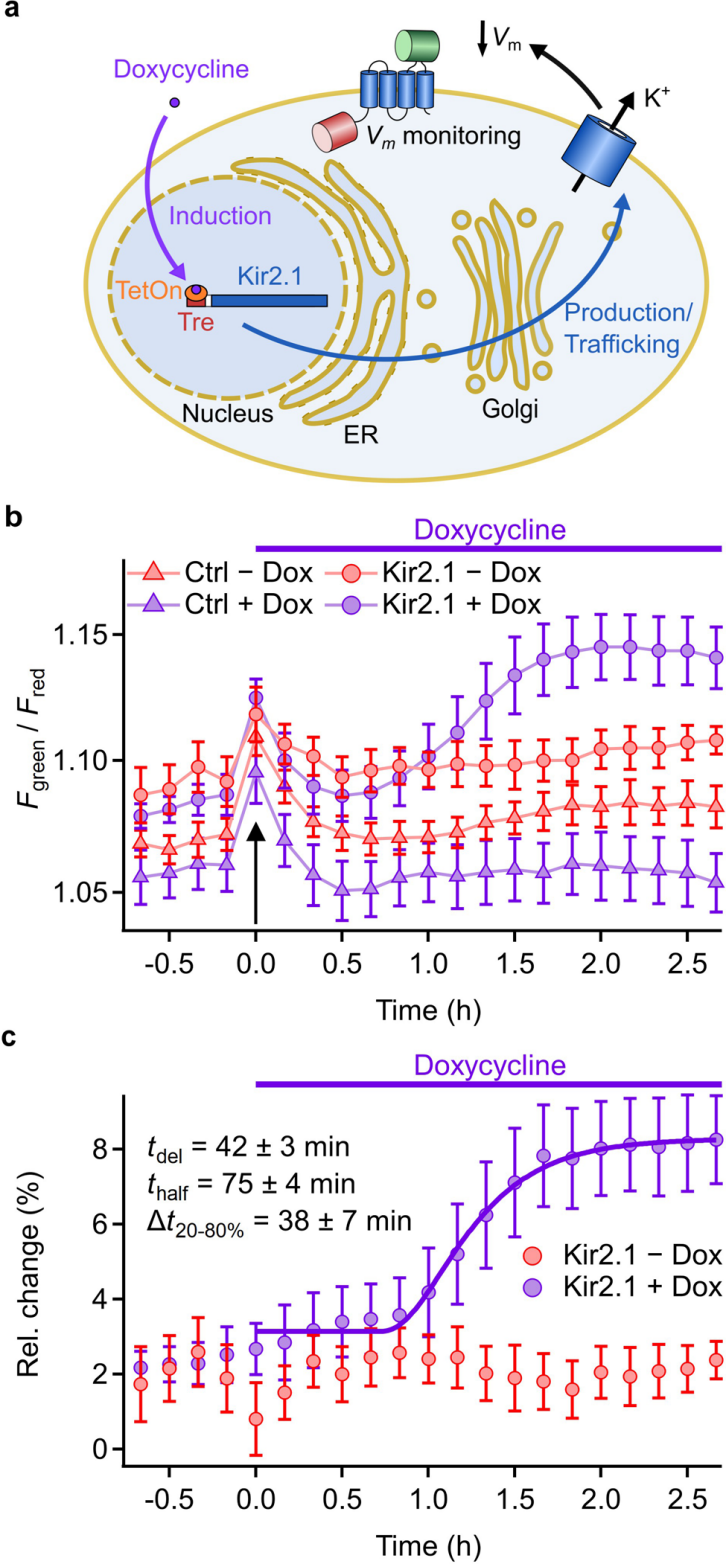
Time course of cell hyperpolarization induced by triggered expression of Kir2.1. **a** Schematic of measurement of production speed of functional Kir2.1 channels using ratiometric GEVIs as *V*_m_ monitor. GEVIs are constitutively produced from an expression vector with CMV promoter. Kir2.1 expression is controlled by the Tre/TetOn system, which is triggered by the application of doxycycline. **b** Time course of *F*_green_/*F*_red_ of HEK293T cells transfected with rASAP and a doxycycline-inducible expression vector system. Gene expression of Kir2.1 or Ctrl (empty pDox vector) with 200 ng/ml doxycycline (Dox) was induced as indicated (purple symbols). Application of solution induced a transient increase of *F*_green_/*F*_red_ in all samples (black arrow). Under control conditions (red symbols), the same application handling was performed without Dox. Images were acquired every 10 min. DMEM-F12 cell culture medium was exchanged with FluoroBrite DMEM 13 h before doxycycline application. Values are means ± sem of images from four different locations in one cell-culture dish. **c** Relative change in *F*_green_/*F*_red_, normalized to the respective control, as a function of time. The solid curve for the Dox-induced data is a fit according to Eq. 5, resulting in 5.1% maximal relative change and the indicated kinetic parameters.

As a proof of concept, we measured the *F*_green_/*F*_red_ dynamics following the induced expression of Kir2.1 channels in real time over many hours. We designed an expression vector (pDox) based on the TetOn 3G system^41^, allowing the doxycycline-induced expression of Kir2.1 channels (Fig. 5a). Control experiments using the same vector system expressing mCherry showed an up to 650-fold increase in *F*_red_ by doxycycline 24 h after induction (Supplementary Fig. 8a, b). A weak basal leak transactivation was seen also in the absence of doxycycline. Accordingly, cells transfected with rASAP and Kir2.1 in the pDox vector showed slightly elevated *F*_green_/*F*_red_ values compared with cells transfected with rASAP and the empty pDox vector (Supplementary Fig. 8c).

Because the cell-culture medium dedicated for live-cell imaging (FluoroBrite DMEM + 10% FCS) exhibited strong green background fluorescence, we bleached the medium for 3 h with 470-nm light before use (Supplementary Fig. 9a, b). The bleached medium by itself neither altered the cell number nor rASAP’s *F*_green_/*F*_red_ of the transfected cells compared to non-bleached medium (Supplementary Fig. 9c–e). We did not use Hoechst 33342 for nuclei identification in long-term live-cell imaging experiments because of its previously reported phototoxicity under repeated illumination^33^; instead, the *F*_red_ and *F*_green_ was integrated for all pixels with *F*_red_ counts between 500 and 3500. Each cell sample was characterized by the mean *F*_green_/*F*_red_ (±sem) of four images at different locations in one cell-culture dish.

HEK293T cells were transfected with rASAP and Kir2.1 in pDox or rASAP and empty pDox vectors (Ctrl). Consistent with the basal transactivation activity of the pDox vector, *F*_green_/*F*_red_ values in the cells transfected with Kir2.1 expression vector were already elevated compared to control cells before application of doxycycline (compare circles with triangles in Fig. 5b). After an initial transient in *F*_green_/*F*_red_ of about two sample points for all cell samples— presumably caused by opening the incubation chamber and mixing the cell-culture media—only the cells with the Kir2.1 expression vector and doxycycline application showed delayed increase in *F*_green_/*F*_red_ over the empty vector controls (Fig. 5b, c), indicating cell hyperpolarization via doxycycline-induced increase in Kir2.1 protein production. The change of around 8% over empty control vector (Fig. 5c) was smaller than in the high-content assay described above (15.9%, Fig. 3g). This is likely attributed to the analysis method, which integrates the signal from the whole cells including GEVI signal originating from non-responsive parts of the cells such as Golgi and ER. The time course of *F*_green_/*F*_red_ followed a sigmoidal saturating function, reaching a half-maximal change in *F*_green_/*F*_red_ after about 73 min with an increase from 20 to 80% within 34 min (Fig. 5c). The initial delay in the change in *F*_green_/*F*_red_ of about 40-50 min provides an estimate of the minimum biogenesis latency of functional Kir2.1 channels in HEK293T cells, including induction of transcription, translation, subunit assembly, and membrane trafficking.

## Discussion

The ratiometric genetically encoded voltage indicators rArc and rASAP were developed for comparison of resting *V*_m_ of different cell groups. rASAP is preferred over rArc based on faster kinetics, pH_i_ stability, and larger dynamic range. With a change in fluorescence-intensity ratio of about 44% in the physiological *V*_m_ range between −100 and 60 mV, rASAP is well suited to resolve both hyperpolarizing and depolarizing events. The high-content workflow protocol (Fig. 2a) allows to detect long-term changes in compound resting *V*_m_ in non-excitable cells caused by overexpression of various K^+^ channel types. The impacts of these channels on the *F*_green_/*F*_red_ values of rASAP are nicely commensurate with the electrophysiological characteristics of the channels. The developed approach, therefore, allows to survey the phenotypes of channel mutants with high throughput without electrode-based electrophysiology. Due to its less invasive nature compared to electrode-based methods, the optical approach allows to compare compound resting *V*_m_ under physiological conditions. Furthermore, ratiometric GEVIs are suited to track the kinetics of functional ion channel biogenesis in real time, which had not been previously achieved. Using rASAP, we estimate the latency for an impact on *V*_m_ by the appearance of functional Kir2.1 channels in the plasma membrane, including induction of transactivation, transcription, translation, subunit assembly, and membrane trafficking, to be about 40–50 min.

With the developed techniques, compound resting *V*_m_ of different cell populations are reliably compared, but the calibration of the automatically acquired signals requires care. Based on calibration parameters derived from patch-clamp experiments with manually selected ROIs encompassing cell edges only, we estimated a resting *V*_m_ in HEK293T cells of roughly −40 mV, similar to those in other studies^10,34^. Assuming a constant *V*_m_ of −40 mV for control HEK293T cells, the estimated *V*_m_ in cells overexpressing Kir2.1 was −95 mV, which is compatible with the equilibrium potential for K^+^ (Fig. 3a), between −90 and −100 mV under live-cell conditions (Supplementary Fig. 10), and Kir2.1 being the major determinant for *V*_m_ under this condition. However, the calculated absolute *V*_m_ values must be interpreted cautiously because the calibration strongly depends on the area of ROI selection and the amount of unresponsive intracellular fractions of the GEVI signal (Supplementary Fig. 10). We observed that the automatic analysis procedure detecting the cell nucleus mainly uses signal of defocused membrane regions from above and below the nucleus and largely excludes areas in the cell with especially bright intracellular structures, which are likely originating from GEVI signal in Golgi (Fig. 2a). In addition, the stringent upper limit removes cells with strongly overexpressed GEVI and thus especially poor membrane trafficking (Fig. 2a, b, Supplementary Fig. 11). Both factors together may explain the unexpectedly good outcome of the automated analysis procedure.

The broad cell-to-cell variation of the *F*_green_/*F*_red_ signal of all developed GEVIs may appear to suggest a large *V*_m_ heterogeneity. However, the *F*_green_/*F*_red_ distribution did not sharpen with gramicidin application, which should bring all cells close to 0 mV and therefore eliminate large *V*_m_ variations among cells. Therefore, the cell-to-cell variation seems to be mainly related to *V*_m_-independent variations of *F*_green_/*F*_red,_ which might be caused by factors as imperfect trafficking as well as differential maturation, degradation, and sensitivity to pH_i_ and the cellular redox state of the two fluorescent components of the sensor. While the broad cell-to-cell variation does not offer single-cell *V*_m_ accuracy, the developed high-content methods clearly allow to detect difference in average resting *V*_m_ among independent cell groups and will be also applicable for existing and future ratiometric GEVIs. Single-cell *V*_m_ accuracy of future ratiometric GEVIs not only requires improvements in Δ*r* but also in plasma membrane trafficking^23^. Furthermore, a single fluorescence component that already provides a ratiometric signal, as in the ratiometric Ca^2+^ indicator ratio pericam^42^, may be beneficial.

Using our newly developed tools we observed that Kv10.1 channels, which are upregulated in 80% of the most common solid tumors^4^, mildly hyperpolarize HEK293T cells when ectopically expressed. The mutation K217N in Kv10.1, which causes severe developmental defects as observed in Temple-Baraitser syndrome patients^6^, shifts average *V*_m_ by roughly −40 mV compared to control HEK293T cells, consistent with the electrophysiological data (Fig. 4; Supplementary Fig. 10). The developmental defects observed in Temple-Baraitser syndrome therefore may be caused by hyperpolarization of mesenchymal stem cells, where Kv10.1 channels are reported to influence cell proliferation and differentation^8^. Missense mutations leading to complex developmental phenotypes have been associated with various K^+^ channel genes and many of the mutations increase channel activity. However, the underlying mechanisms are poorly understood^3^. Long-lasting cell hyperpolarization may be a common feature for some of the disease-causing mutations including mutants of *KCNJ*2, *KCNJ*6, *KCNJ*8, and *KCNN*3^3^. Studying such mutants with the devised methods for their impact on *V*_m_ will advance our knowledge on mechanistic roots of such developmental diseases and the underlying regulatory processes.

An important prerequisite to a wide application is the feasibility of applying rASAP in various cell types. As indicated in Supplementary Fig. 4, voltage-dependent rASAP signals are readily detectable in the neuroblastoma and melanoma cells examined. Since the fraction of *V*_m_-responsive and *V*_m_-unresponsive components of GEVI fluorescence may vary between different cell types, cell-specific calibration is required.

In conclusion, the ratiometric *V*_m_ sensors and the data acquisition and analysis techniques described here are well suited for detecting differences in compound *V*_m_ among independent cell groups in the whole physiological voltage range and represent a useful set of tools for studying disease-relevant ion channels and variants. The methodologies are applicable for high-throughput ion channel drug screening and should facilitate our understanding of the roles of resting *V*_m_ in fundamental processes such as ion channel biogenesis, transmembrane transport, cell cycle progression, and cancer.

## Methods

### Molecular biology

rArc and rASAP variants were constructed with standard molecular biology methods. Construction of rArc-mCherry: mCherry from pmCherry_C2, extracted with *Nhe*I and *Eco*RI, was combined with PCR-amplified ArcLightQ239 (36856, Addgene), flanked by *Eco*RI and *Not*I restriction sites in pcDNA3.1(+), cut with *Nhe*I and *Not*I (NEB, Ipswich, Massachusetts, USA). rArc-mCherry-M71T was generated by site-directed mutagenesis of mCherry in rArc-mCherry. Analogously, rArc was generated by exchanging mCherry with PCR-amplified mKate2 from heme sensor 1 (159169, Addgene, Watertown, Massachusetts, USA) using flanking *Nhe*I and *Eco*RI restriction sites. rArci, a rArc variant with mutations R229Q and R232Q, was generated by site-directed mutagenesis. Construction of rASAP: ArcLightQ239 was replaced in rArc by an ASAP-coding synthetic DNA double strand (Eurofins Genomics, Luxemburg), which contained flanking *Eco*RI (5′) and *Not*I (3′) sites. Our version of ASAP contained the R415Q mutation of ASAP2s, I67T, and Q397R, which reversed the point mutation introduced in ASAP1^28^. For rASAP-al, the linker between segment S3 and the cpGFP domain of rASAP was exchanged according to Fig. 1b^16^.

For the generation of coexpression vectors, the Kan/Neo gene of pCDNA3.1(+) was replaced with the coding sequences of the K^+^ channels human Slo1 (BK, *KCNMA*1, NP_002238.2), human Kv10.1 (hEAG1, *KCNH*1, NP_002229.1), or mouse Kir2.1 (*Kcnj*2, NP_032451.1). Point mutations were introduced by site-directed mutagenesis.

The doxycycline-inducible vector (here called pDox) was generated by exchanging the CMV promoter of pCDNA3.1(+) with the TRE3G promoter and the Kan/Neo cassette with the coding sequence for the TetOn3G gene, of pHR-EF1Alpha-puro-T2A-Tet-on 3G-TRE3G-Ascl1 (118593, Addgene). For inducible expression of Kir2.1 or mCherry, the corresponding genes were placed in the multiple-cloning site of the pDox vector following the Tre promoter. All clones were verified with DNA sequencing (Eurofins Genomics). Sequences of plasmids used in this study are available as supplementary information files (Supplementary Data 3).

### Cell culture

Human embryonic kidney 293T cells (HEK293T; CAMR, Porton Down, Salisbury, UK) were cultured in 1:1 Dulbecco’s Minimal Eagles Medium and Ham’s F12 medium (DMEM-F12, Thermo Fisher Scientific, Waltham, Massachusetts, USA) supplemented with 10% fetal bovine serum in a humidified incubator at 37 °C with 5% CO_2_. Mouse neuroblastoma Neuro2A cells (DSMZ, Braunschweig, Germany) were cultured in Minimal Eagles Medium (MEM, Sigma Aldrich) and human melanoma A375 cells (ATCC, Manassas, VA, USA) were cultured in DMEM (Sigma Aldrich) with 10% fetal bovine serum (FCS) in a humidified incubator at 37 °C with 10% CO_2_.

For electrophysiological, high-content, and long-term live-cell imaging experiments, cells were plated on 35-mm glass-bottom dishes (Ibidi, Martinsried, Germany) at ≈20,000 cells/dish. One day after plating, HEK293T and Neuro2A cells were transfected with plasmids using the Roti®-fect transfection kit (Carl Roth, Karlsruhe, Germany) with 1 µg DNA per glass-bottom dish. A375 cells were electroporated (4D-Nucleofector, Lonza, Basel, Switzerland) with 1.5 µg DNA using the SF Cell Line 4D-Nucleofector kit (Lonza) according to the supplier instructions.

Electrophysiological and high-content recordings were performed two days after transfection for HEK293T cells and one day after transfection for Neuro2A and A375 cells.

### Imaging and photometry under electrophysiological control

Combined electrophysiological and imaging recordings (Supplementary Fig. 1) were performed on an Axio Observer inverted microscope equipped with the Colibri-2 LED illumination system (Carl Zeiss, Jena, Germany) and an EC-Plan Neofluar oil 40× objective (NA 1.3, Zeiss). Images were acquired with a CMOS camera (ORCA-Flash 4.0 Digital Camera C11440, Hamamatsu, Japan) operated with SmartLux software (HEKA Elektronik, Lambrecht, Germany). Electrophysiological measurements were performed with an EPC10 double patch-clamp amplifier controlled by PatchMaster software (HEKA Elektronik). The following excitation light sources were used: 470-nm LED (BP 460/50) for ASAP and ArcLight, 530-nm LED (BP 545/40) for mCherry and mKate2. Excitation and emission light were passed through an EGFP/mCherry (59022 Chroma) dual-emission filter set.

For experiments requiring high temporal resolution (Supplementary Fig. 2), patch clamp was combined with photometry. Light from a 470-nm LED (M470L1, Thorlabs), an EC-Plan Neofluar oil-immersion 40× objective (1.3 NA, Zeiss), and an EGFP/mCherry dual-emission filter were used. Emitted light was measured with a photodiode (FDU photodiode with view finder, T.I.L.L. Photonics, Gräfelfing, Germany) and recorded with PatchMaster (HEKA Elektronik).

Patch-clamp pipettes were fabricated from borosilicate glass, coated with dental wax and fire polished to resistances of 1–2 MΩ. Prior to recordings, the cell culture medium was removed from cells and exchanged with external solution containing (in mM) 146 NaCl, 4 KCl, 2 CaCl_2_, 2 MgCl_2_, and 10 HEPES (adjusted with NaOH to pH 7.4 at 22 °C). The pipette solution contained (in mM) 130 KCl, 2.5 MgCl_2_, 10 ethylene glycol tetraacetic acid (EGTA), and was adjusted to pH 6.9, 7.4, or 7.9 at 22 °C with KOH. Currents were low-pass filtered at 5 kHz and sampled at 20 kHz.

### Live-cell fluorescence imaging

Imaging experiments were performed on an Eclipse-Ti fluorescence microscope equipped with a DS-Qi2 camera (14 bit; Nikon, Tokyo, Japan) and a X-Cite 120 LED light source (Excelitas Technologies, Waltham, Massachusetts, USA), all controlled by the NIS4.6 software (Nikon).

The temperature of the incubation chamber (Oko Lab, Pozzuoli, Italy) was adjusted to 37 °C before the start of the measurement. Before measurements, cell culture medium was removed, the cells were washed once with 1 ml measurement buffer (see external solution for patch-clamp experiments), and subsequently the medium was replaced with 2 ml measurement buffer supplemented with 5 mM glucose and 10 µg/ml Hoechst 33342 (Invitrogen). Cells were then incubated for 30 min at 37 °C and 5% CO_2_ before image acquisition. BaCl_2_ was dissolved in the measurement buffer. Gramicidin D (G5002, Sigma-Aldrich) was dissolved at 2 mM in ethanol and diluted to 1 µM in measurement buffer immediately before experiments. DCPIB (4-(2-butyl-6,7-dichloro-2-cyclopentyl-indan-1-on-5-yl) oxobutyric acid (Cruz Chem, Santa Cruz Biotechnology, Santa Cruz, CA, USA) was dissolved at 10 mM in DMSO and diluted to 10 µM in measurement buffer immediately before use.

For every 35-mm glass-bottom dish, images from 16 spots were acquired using a 10× objective (Plan Apo λ, NA 0.45, Nikon) and an automated stage (H117 stage with a Proscan III controller; Prior Scientific, Cambridge, UK) controlled by the NIS 4.6 (Nikon) software, avoiding an overlap of illuminated areas. The z-focus was stabilized with the Nikon Perfect Focusing System. Four images were taken with blue excitation (GFP-3035D-000, 0.5 or 1.0 s exposure time each with a camera gain factor of 5.1) and one with green excitation (4040C-000 BrightLine, 0.5 or 1.0 s exposure time, with a camera gain factor of 5.1). The Hoechst 33342 signal was recorded with UV excitation (DAPI-50LP-A-000, 3 ms; filter sets from Semrock, Rochester, NY, USA). In addition, one image was acquired in transmission mode.

For real-time live-cell experiments, DMEM-F12 was replaced with FluoroBrite DMEM medium (Thermo Fisher Scientific) supplemented with 10% FCS 24 h after transfection; the medium was bleached for 3 h with a 470 nm LED (100%, M470L1, Thorlabs) before usage (Supplementary Fig. 9). For induction of gene expression from pDox, the medium was supplemented with 200 ng/ml doxycycline hyclate dissolved at 1 mg/ml in water (Sigma-Aldrich). Recordings were performed in a humidified atmosphere at 37 °C and 5% CO_2_. To minimize photobleaching, one green and one red image (200 ms exposure each, camera gain factor of 13.9) were acquired every 10 min.

### Automated image analysis

Image analysis was performed with Fiji software^43^. For high-content imaging, the camera offset for each channel was subtracted; the background of each image was subtracted individually using the built-in rolling-ball algorithm (radius of 146 μm). Subsequently, cell nuclei were detected in the Hoechst 33342/blue channel by converting the image into a binary mask with a manually set threshold. The built-in watershed algorithm was then used to separate nuclei that were too close to be separated via thresholding. Using the particle analysis tool of Fiji, individual nuclei were detected for each image. Particles crossing the edge of the image, as well as particles of unreasonable size (<26 μm^2^ and >117 μm^2^) were excluded. ROIs were automatically drawn at the boundary of each particle, and fluorescence parameters were extracted for each individual cell for the green, red, and blue channels. For the green channel, only the fourth image was used for analysis in order to measure *F*_green_ after complete photoswitching (Supplementary Fig. 1a). A schematic illustration of the analysis procedure is shown in Fig. 2a. Compiled data were further analyzed with Igor Pro 8 (WaveMetrics, Lake Oswego, OR, USA).

Several measures were applied to avoid bias due to technical limitations. First and foremost, the limited dynamic range of the recording camera defines an upper limit for strongly expressing cells and high *F*_green_/*F*_red_ values, which systematically excludes high *F*_green_/*F*_red_ values (Supplementary Fig. 11). Thus, cells with very high *F*_red_ signals were excluded from analysis (Fig. 2b). Second, insufficient background subtraction can result in systematic, expression-dependent changes in *F*_green_/*F*_red_. This problem was especially conspicuous for *F*_green_ when the strongly auto-fluorescent cell culture medium (DMEM-F12) was not completely removed (Supplementary Fig. 12). Therefore, the median *F*_green_/*F*_red_ values as a function of *F*_red_ were plotted and a linear fit was used to determine the minimum of the squared slope (slope^2^) as a function of background *F*_green_ (Supplementary Fig. 12). In addition, cells with low *F*_red_ intensities, for which the error in background subtraction was largest, were excluded. The remaining cells were gated according to the cell-cycle stage: cells in the G0/G1, S, and G2/M phase were used for analysis, while cells with signals below the G0/G1 peak were considered as debris, and cells above the G2/M peaks as non-separated doublet signals.

For live-cell real-time experiments the image background for both channels was subtracted with the built-in rolling-ball algorithm. The reported phototoxicity of Hoechst 33342 under repeated illumination^33^ excluded its use for long-term experiments. Instead, we used the *F*_red_ signal for generating a binary mask (500 counts < *F*_red_ < 3500 counts) and exported the average *F*_green_ and *F*_red_ values from the selected area of each image.

Fluorescence ratio–*V*_m_ curves were fit according to the following Boltzmann-type function:

1 $$\frac{{F}_{{{{{\mathrm{green}}}}}}}{{F}_{{{{{\mathrm{red}}}}}}}({V}_{m})={R}_{\max }\left\{1-\frac{\varDelta r/100}{1+{e}^{-({V}_{m}-{V}_{{{{{\mathrm{half}}}}}})/{k}_{s}}}\right\}$$

with the maximum fluorescence ratio *R*_max_, the maximal relative change in ratio (Δ*r*, in %), the voltage of half-maximal fluorescence change, *V*_half_, and *k*_s_ characterizing the steepness.

Kinetics of fluorescence change by voltage steps were analyzed with double-exponential fits:

2 $$\frac{{F}_{{{{{\mathrm{green}}}}}}}{{F}_{\max }}(t)=1-\frac{\varDelta r}{100}[{a}_{{{{{\mathrm{fast}}}}}}(1-{e}^{-t/{\tau }_{{{{{\mathrm{fast}}}}}}})+{a}_{{{{{\mathrm{slow}}}}}}(1-{e}^{-t/{\tau }_{{{{{\mathrm{slow}}}}}}})]$$

with the relative amplitudes *a*_fast_ + *a*_slow_ = 1 and the time constants *τ*_fast_ and *τ*_slow_.

Potassium equilibrium potential (*E*_K_) was calculated according to the Nernst equation:

3 $${E}_{K}=\frac{RT}{F}\,{{{{\mathrm{ln}}}}}\left(\frac{{[{K}^{+}]}_{{{{{\mathrm{out}}}}}}}{{[{K}^{+}]}_{{{{{\mathrm{in}}}}}}}\right)$$

with R = 8.314 J K^−1^mol^−1^, *T* = 310.15 K and *F* = 96,485 C mol^−1^; [K^+^]_in_ and [K^+^]_out_ are internal and external K^+^ concentrations, respectively.

Frequency distributions of *x* = log(*F*_green_/*F*_red_) were described with Gaussian functions:

4 $$f(x)={f}_{\max }{e}^{-\frac{{(x-{x}_{0})}^{2}}{2{\sigma }^{2}}}$$

with the maximal amplitude *f*_max_, the center of the distribution *x*_0_, and the standard deviation σ.

The time course in *F*_green_/*F*_red_ following the induced production of ion channels was fit with a sigmoidal function for *t* > 0, from which the time at half-maximal change and the time for 20–80% change were analytically derived:

5 $$\frac{{F}_{{{{{\mathrm{green}}}}}}}{{F}_{{{{{\mathrm{red}}}}}}}(t)={y}_{0}+({y}_{\max }-y{}_{0}){\left(1-{e}^{-(t-{t}_{{{{{\mathrm{del}}}}}})/\tau }\right)}^{3}$$

with initial offset *y*_0_, the maximal relative ratio *y*_max_, an initial delay *t*_del_, and a time constant τ.

### Statistics and reproducibility

Data are typically depicted as means ± sem or median values unless stated otherwise. Boxes of boxplots feature 25/75% quantiles of the distributions, whiskers are 10/90% quantiles, and the central line is the median. The standard deviation of the median from individual *F*_green_/*F*_red_ distributions was estimated with resampling with replacement (Igor Pro 8). For small sample sizes, all individual data points are presented. The number of individual measurements is referred to as *n* and the number of independent replicates on different days as *N*.

### Reporting summary

Further information on research design is available in the Nature Research Reporting Summary linked to this article.

## Supplementary information

Supplementary Information

Description of Additional Supplementary Files

Supplementary Data 1

Supplementary Data 2

Supplementary Data 3

Reporting Summary

## Data Availability

All data generated or analyzed during this study are included in this published article and its supplementary information files (Supplementary Data 1–2). Plasmids of the sensor constructs are available through Addgene. Any remaining information can be obtained from the corresponding author upon reasonable request.
