# Supplementary material for: Monitoring of compound resting membrane potentials of cell cultures with ratiometric genetically encoded voltage indicators
Source: Commun Biol. 2021 Oct 7;4:1164. doi: 10.1038/s42003-021-02675-0 (PMC8497494; doi:10.1038/s42003-021-02675-0)
Supplement: Supplementary file 1 — Supplementary Information [file 42003_2021_2675_MOESM1_ESM.pdf]

Monitoring of compound resting membrane potentials of cell cultures with ratiometric genetically encoded voltage indicators

*Philipp Rühl[1], Johanna M. Langner[1], Jasmin Reidel[1], Roland Schönherr[1], Toshinori Hoshi[2], Stefan H. Heinemann[1]**

1. Center for Molecular Biomedicine, Department of Biophysics, Friedrich Schiller University Jena and Jena University Hospital, D-07745 Jena, Germany

2. Department of Physiology, University of Pennsylvania, Philadelphia, Pennsylvania, United States of America

* Corresponding author

Supplementary Material

Supplementary Tables

Supplementary Table 1. Parameters (mean ± sem, *n* in parentheses) describing the V_m dependencies of F_{green}/F_{red} as a result of fits according to Eq. (1), as shown in **Fig. 1**.

GEVI (*n*)	pH	R_{max}	Δr (%)	V_{half} (mV)	k_s (mV)
rArc (11)	6.9	0.96 ± 0.04	44 ± 3	-64.1 ± 1.5	30.3 ± 0.5
rArc (10)	7.4	1.87 ± 0.06	41.2 ± 1.6	-48.7 ± 1.8	27.8 ± 0.5
rArc (7)	7.9	2.73 ± 0.13	33 ± 3	-26 ± 3	27.1 ± 1.4
rASAP (10)	6.9	2.01 ± 0.10	61 ± 4	-11 ± 3	55.4 ± 2.2
rASAP (10)	7.4	1.71 ± 0.05	62.6 ± 2.3	-11.4 ± 1.7	53.8 ± 1.4
rASAP (10)	7.9	1.60 ± 0.09	52 ± 4	-14.2 ± 1.9	51.9 ± 0.9
rASAP-al (11)	7.4	1.89 ± 0.09	60 ± 5	19 ± 3	36.7 ± 0.8

Supplementary Figures

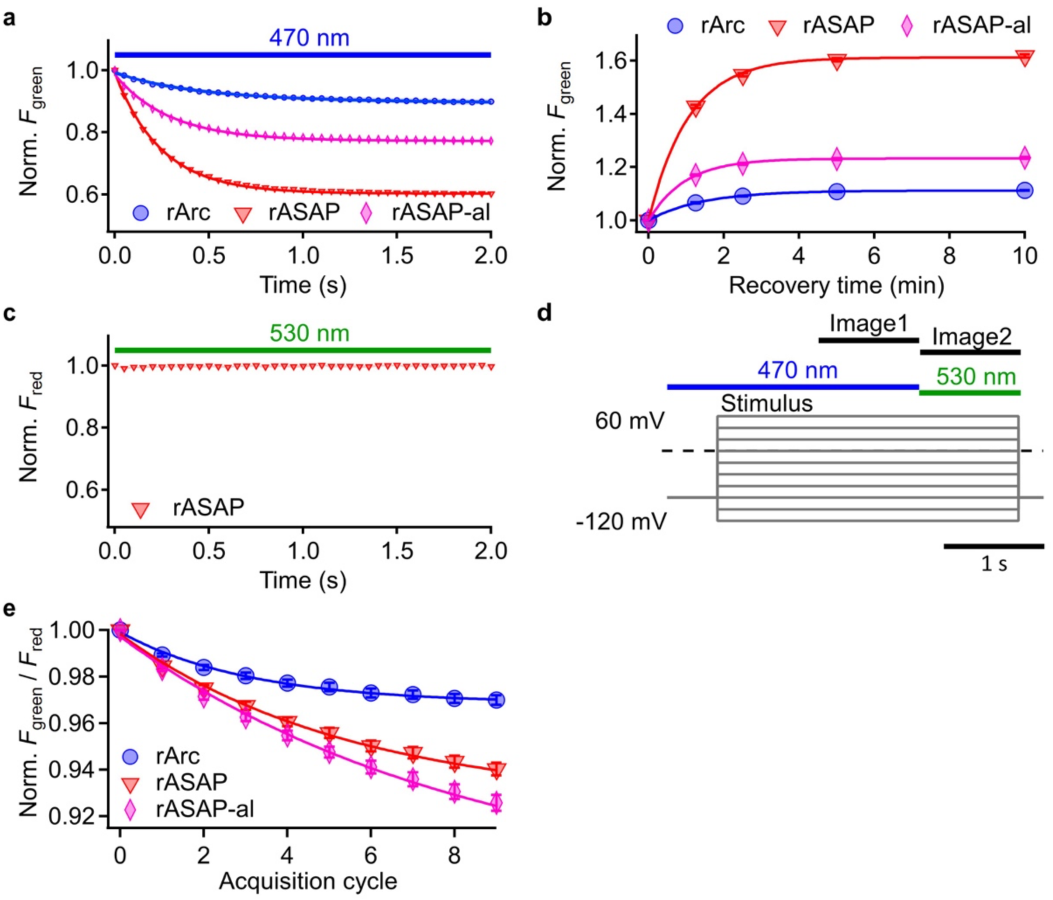

Supplementary Figure 1 | Photoswitching and photobleaching of rArc, rASAP and rASAP-al. (**a**) All GEVIs undergo rapid loss of their green fluorescence after start of illumination. Data are normalized F_{green} signals as a function of illumination duration (100% of 470-nm LED with a 40x objective) for rArc, rASAP, and rASAP-al expressed in HEK293T cells. Photoswitching time constants and their relative amplitudes were determined with single exponential fits: 510 ± 3 ms, 9.5 ± 0.7% (rArc); 248 ± 5 ms, 38.9 ± 0.4% (rASAP); 293 ± 4 ms, 21.37 ± 0.16% (rASAP-al); n = 10 cells each. (**b**) The loss of fluorescence is reversible: recovery kinetics of F_{green} after photoswitching was driven into saturation with the protocol of **a**. Solid curves are single-exponential fits; recovery time constants at 23 °C were 87 ± 3 s (rArc), 63.8 ± 1.4 s (rASAP), 58.2 ± 1.7 s (rASAP-al), n = 10-15 cells each. (**c**) The photoswitching behavior was not observed for mKate2 (100% of 530-nm LED with a 40x objective); n = 10 cells. The F_{red} signal is exclusively shown for rASAP for clarity. (**d**) To drive the photoswitching of F_{green} into saturation before green (Image 1) and red fluorescence images (Image 2) were taken, the pulse protocol used for fluorescence-V_m measurements included a 1.5 s 470-nm pre-illumination. The voltage pulse protocol was used for fluorescence-V_m calibration curves in **Fig. 1**. (**e**) Due to the long illumination protocol in **d**, the F_{green}/F_{red} signal decreases over time mainly by photobleaching of F_{green}. Data are normalized F_{green}/F_{red} values as a function of acquisition cycle of resting cells without electrophysiological control using the illumination and image acquisition protocol from **d** (repetition rate: 0.2 Hz). The decrease in F_{green}/F_{red} was fit with single exponentials yielding the following time constants and relative changes in

F_{green}/F_{red}: 3.5 ± 0.4 cycles, 3.3 ± 0.3% (rArc), 6.9 ± 0.8 cycles, 7.8 ± 0.4% (rASAP), and 8.7 ± 0.3 cycles, 11.3 ± 0.5% (rASAP-al); n = 42-50 cells in total from 10 separate areas. The time courses were used to correct $F_{green}/F_{red}(V)$ data traces in **Fig. 1**. Data are means ± sem.

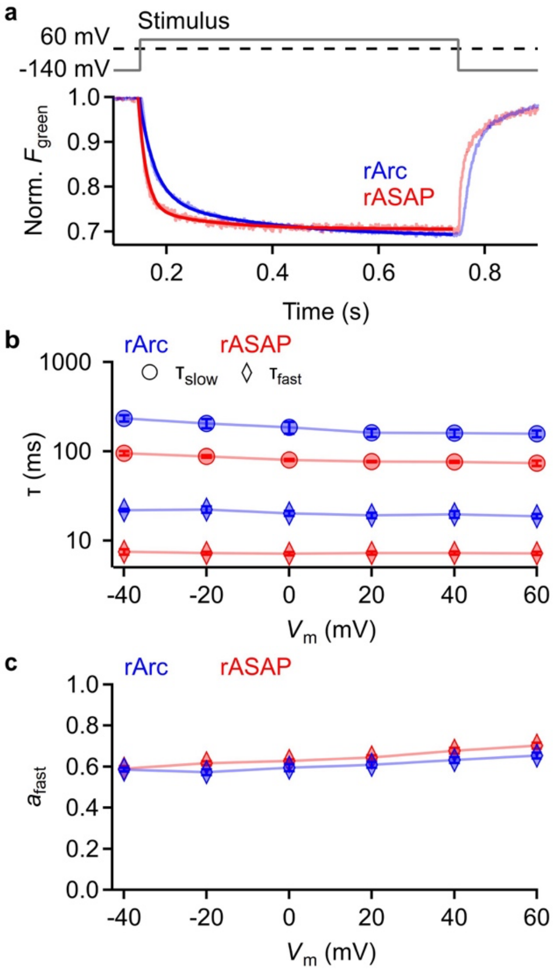

Supplementary Figure 2 | Kinetics of F_{green} for rArc and rASAP. (**a**) Representative photometry measurement of the normalized F_{green} signal of HEK293T cells expressing rArc (blue) or rASAP (red) voltage-clamped according to the indicated protocol at 23 °C. F_{green} photoswitching was driven into saturation before measurement. The F_{green} response to depolarization was fit with a double-exponential function (Eq. 2); fits are superimposed. (**b**,**c**) Fast and slow time constants (**b**) and relative amplitudes (a_{fast}) of the fast component (**c**) resulting from double-exponential fits (as in **a**) as a function of V_m; n = 8-9; data are means ± sem; straight lines connect data points for clarity.

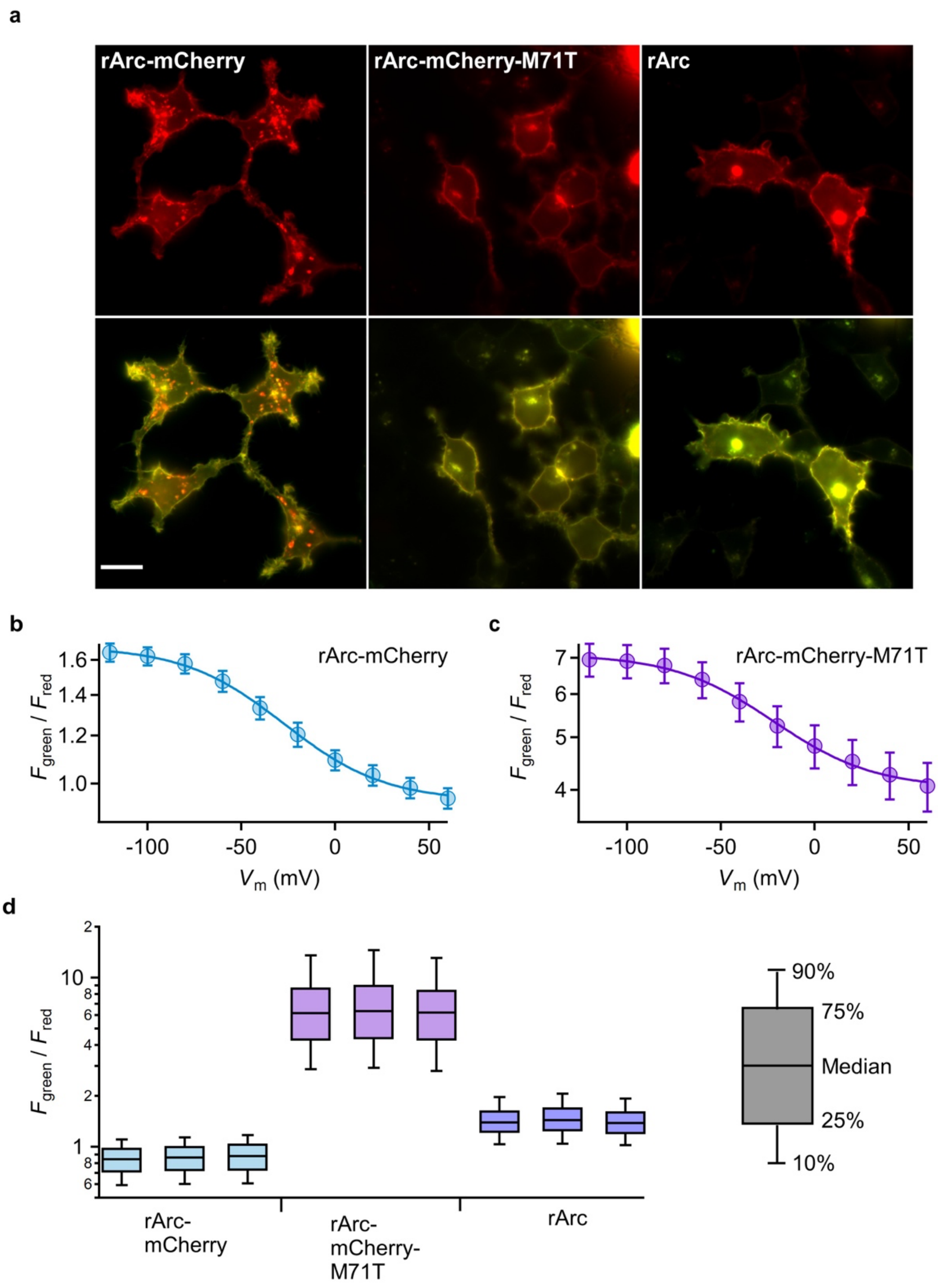

Supplementary Figure 3 | rArc constructs based on mCherry. (**a,** *top*) F_{red} and (**a,** *bottom*) F_{green} and F_{red} composite images of HEK293T cells expressing rArc-mCherry, rArc-mCherry-M71T or rArc, taken 2 days after transfection. Scale bar, 20 µm. The F_{red} channel shows intense red fluorescent small punctate structures in cells expressing rArc-mCherry, which are absent in cells transfected with rArc-mCherry-M71T or rArc. (**b**) F_{green}/F_{red} as a function of V_m for images of voltage-clamped HEK293T cells expressing rArc-mCherry (n = 32); Boltzmann-type functions (Eq. 1) are superimposed. (**c**) As in **b** but with cells expressing rArc-mCherry-M71T (n = 9). Pulse protocol is shown in **Supplementary Fig. 1d**; data are means ± sem. Parameters of Boltzmann fits were R_{max} = 1.68 ± 0.05, Δr = 43.9 ± 1.6, V_{half} = -37.9 ± 2.1 mV, k_s = 25.3 ± 0.7 mV (rArc-mCherry) and R_{max} =

7.1 ± 0.5, Δr = 43.2 ± 3.2, V_{half} = -30 ± 3 mV, k_s = 25.7 ± 0.6 mV (rArc-mCherry-M71T). (**d**) rArc- mCherry-M71T has a smaller F_{red} signal and an increased cell-to-cell variation in the F_{green}/F_{red} signal compared to rArc-mCherry and rArc. (*left*) Each boxplot features the cell-to-cell variations of the F_{green}/F_{red} signal of HEK293T cells expressing the respective GEVI on an individual cell-culture dish; results from 3 independent cell-culture dishes are shown with > 3400 cells, each. Data were acquired with the high-content V_m assay described in **Fig. 2a**. (*right*) Description of the boxplot parameters.

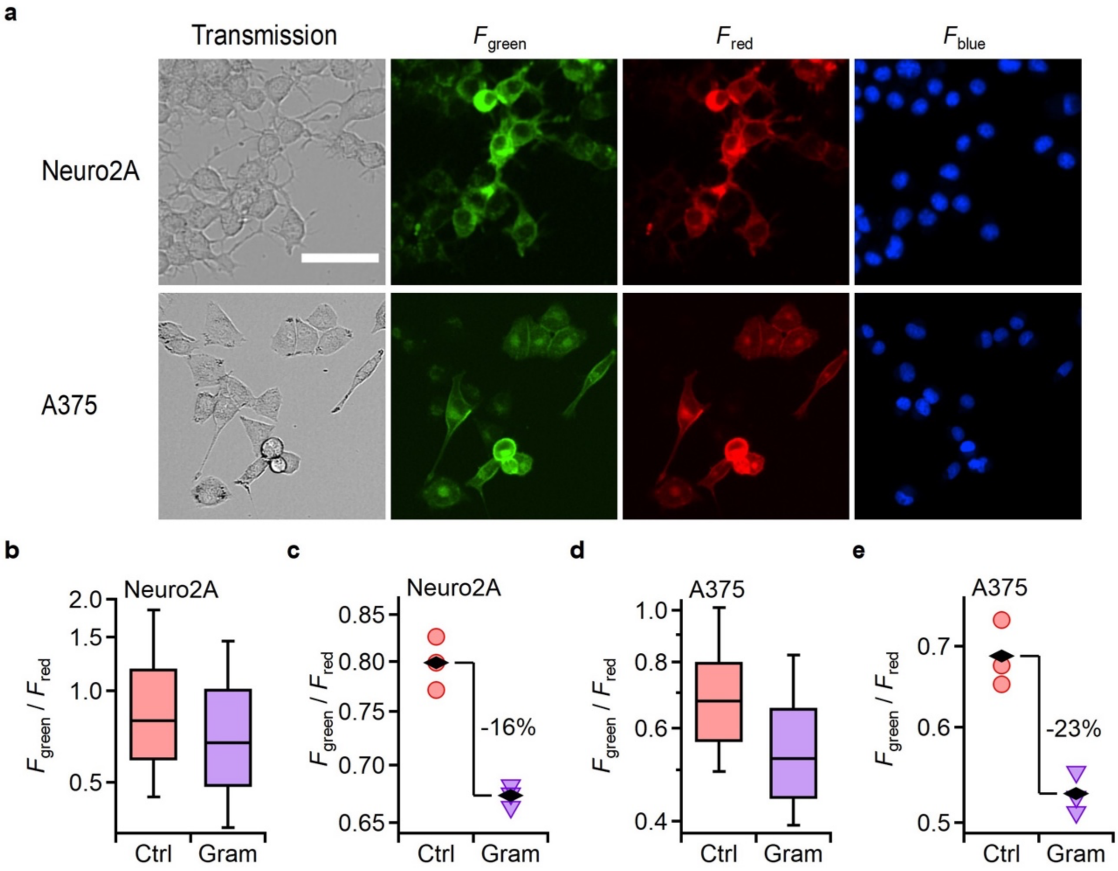

Supplementary Figure 4 | Expression of rASAP in cancer cell lines. (**a,** *top*) Images of the mouse neuroblastoma-derived Neuro2A cell line and (**a,** *bottom*) the human melanoma-derived A375 cell line transfected with the rASAP expression vector. Scale bar, 50 µm. F_{green} and F_{red} images depict the signal originating from rASAP. F_{blue} fluorescence is from nuclei stained with the DNA dye Hoechst 33342. (**b**) Cell-to-cell distribution of the F_{green}/F_{red} signal from Neuro2A cells under resting conditions (Ctrl) or treated with the ionophore gramicidin (Gram). All data points for each condition are from cells on a single cell-culture dish. Data were acquired with the high-content V_m assay described in **Fig. 2**. (**c**) Median F_{green}/F_{red} values of rASAP in Neuro2A cells under resting conditions (Ctrl) or treated with gramicidin (Gram). Each data point is the median F_{green}/F_{red} of cells in an individual cell-culture dish. Black rhombi are means of the median F_{green}/F_{red} from the individual cell-culture dishes. Relative change in F_{green}/F_{red} of gramicidin-treated cells compared to control cells is given. For each sample: cells > 1000. (**d, e**) As in **b** and **c** but with rASAP expressed in A375 cells. For each sample: cells > 480.

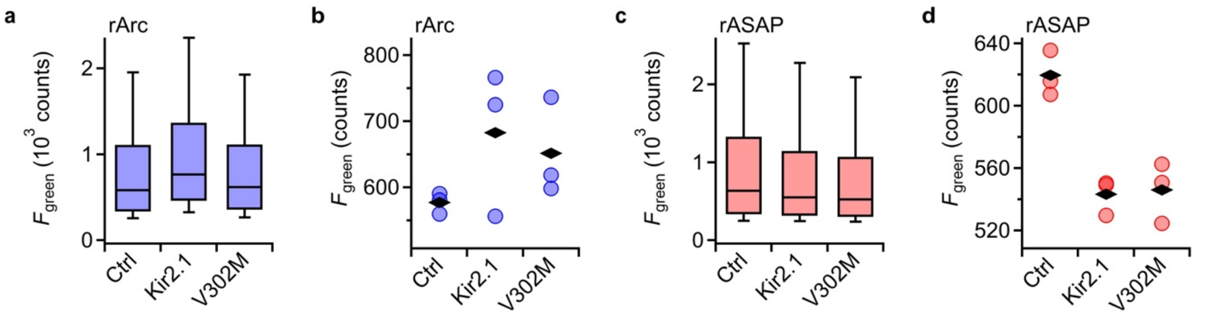

Supplementary Figure 5 | F_{green} alone is insufficient for reliable membrane potential estimation. (**a**) Boxplots of F_{green} from images of HEK293T expressing rArc alone, coexpressing Kir2.1, or Kir2.1-V302M. Lower gate for F_{green} was set to 200 counts to exclude non-transfected cells; cells with F_{green} values close to the upper camera detection limit were also excluded. (**b**) Median F_{green} values of 3 independent cell-culture dishes measured on the same day. Black rhombi indicate means. (**c**, **d**) As in **a** and **b** but with cells expressing rASAP. Measurements were identical to data shown in **Fig. 3b**, **c**, **e**, **f**. For each sample: cells > 3500.

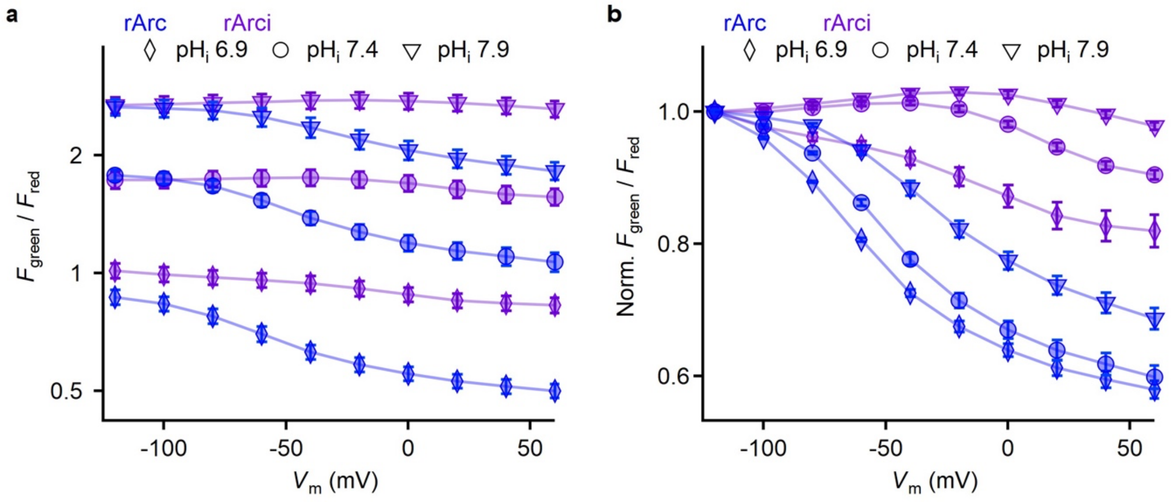

Supplementary Figure 6 | rArci, a rArc variant with diminished voltage sensitivity. (**a**) F_{green}/F_{red} for the indicated intracellular pH values as a function of V_m of voltage-clamped HEK293T cells expressing rArc or rArci, an rArc variant with the mutations R229Q and R232Q in the S4 segment of the voltage-sensing domain of the voltage-sensitive phosphatase of rArc. The pulse protocol is shown in **Supplementary Fig. 1d**; data are means ± sem, n = 9-10 each. rArc data from **Fig. 1** are shown for comparison. (**b**) F_{green}/F_{red} data normalized to the values at -120 mV of **a** as a function of V_m; straight lines connect data points for clarity.

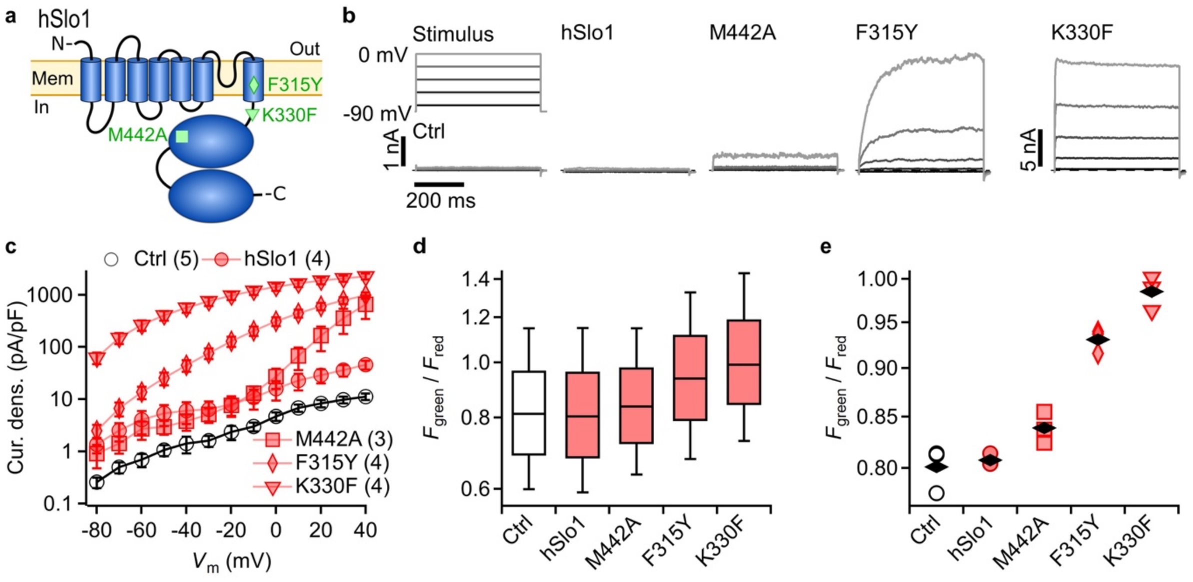

Supplementary Figure 7 | Cell hyperpolarization by hSlo1 gain-of-function mutants. (**a**) Schematic of one of four subunits forming a functional human hSlo1 channel and locations of the point mutations used to increase channel open probability by left-shifting the V_m dependence of channel opening. (**b**) Representative whole-cell current traces of HEK293T cells expressing rASAP alone (Ctrl) or coexpressing hSlo1 or one of the indicated mutants. Current traces were acquired using the depicted V_m pulse protocol. The intracellular solution was nominally Ca^{2+}-free, buffered with EGTA. (**c**) Current density as a function of V_m of cells expressing rASAP alone (Ctrl) or coexpressing one of the indicated hSlo1 variants. Data points are means ± sem, *n* is given in parentheses; straight lines connect data points for clarity. (**d**) Boxplots of F_{green}/F_{red} for HEK293T cells expressing rASAP alone or coexpressing hSlo1 variants; the high-content V_m assay was performed as described in the main text. (**e**) Median ratio values of 3 independent cell-culture dishes (as in **d**) measured on the same day; cells > 6100 each; black rhombi are average values.

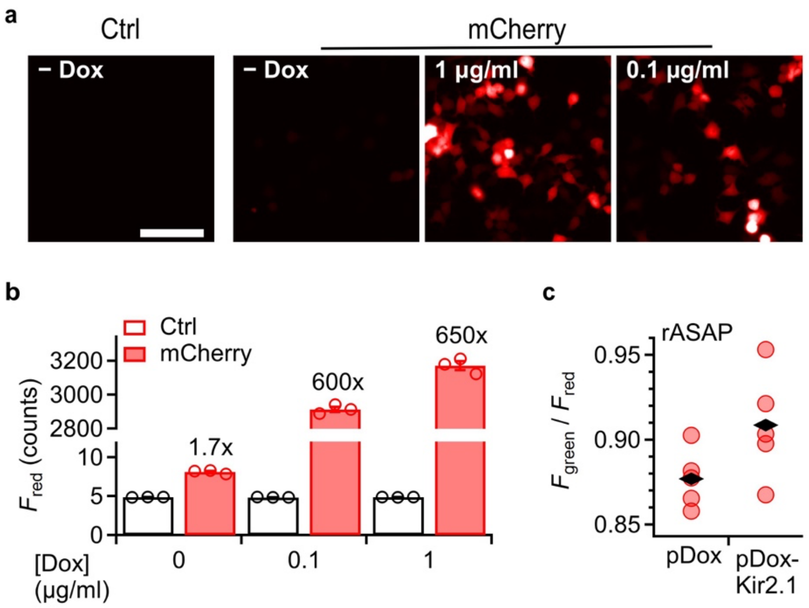

Supplementary Figure 8 | Functional validation of the pDox expression vector. (**a**) F_{red} images of HEK293T cells transfected with pDox or mCherry-pDox expression vectors and treated with the indicated concentrations of doxycycline (Dox). Scale bar, 100 µm. In the expression vector, the coding sequence of mCherry was placed downstream of the tetracycline-responsive element (Tre). Cell-culture medium was exchanged 24 h after transfection with medium containing the indicated amounts of doxycycline. Images were acquired 24-25 h after induction. (**b**) F_{red} of cells transfected without (Ctrl) or with mCherry-pDox (mCherry), treated with 0, 0.1 or 1 µg/ml doxycycline. Data are means ± sem of 3 individual cell-culture dishes for each condition; cells > 7900 each. Circles mark results of individual experiments. Data were acquired and analyzed as shown in **Fig. 2** with adapted exposure times. All samples were cotransfected with super-folder GFP to identify transfected cells. A basal level of mCherry expression is also seen in the absence of doxycycline. Numbers above the bars indicate average fold increase of transactivation compared to empty-vector control cells. (**c**) F_{green}/F_{red} of cells transfected with the rASAP expression vector and the Kir2.1-pDox vector or the empty-vector control. The data points are median F_{green}/F_{red} values of HEK293T cells from five individual cell-culture dishes for each condition one day after transfection with 0.75 µg rASAP expression vector and 0.25 µg pDox or Kir2.1-pDox. Data were acquired and analyzed as shown in **Fig. 2**. Black rhombi are means of median values from individual cell-culture dishes.

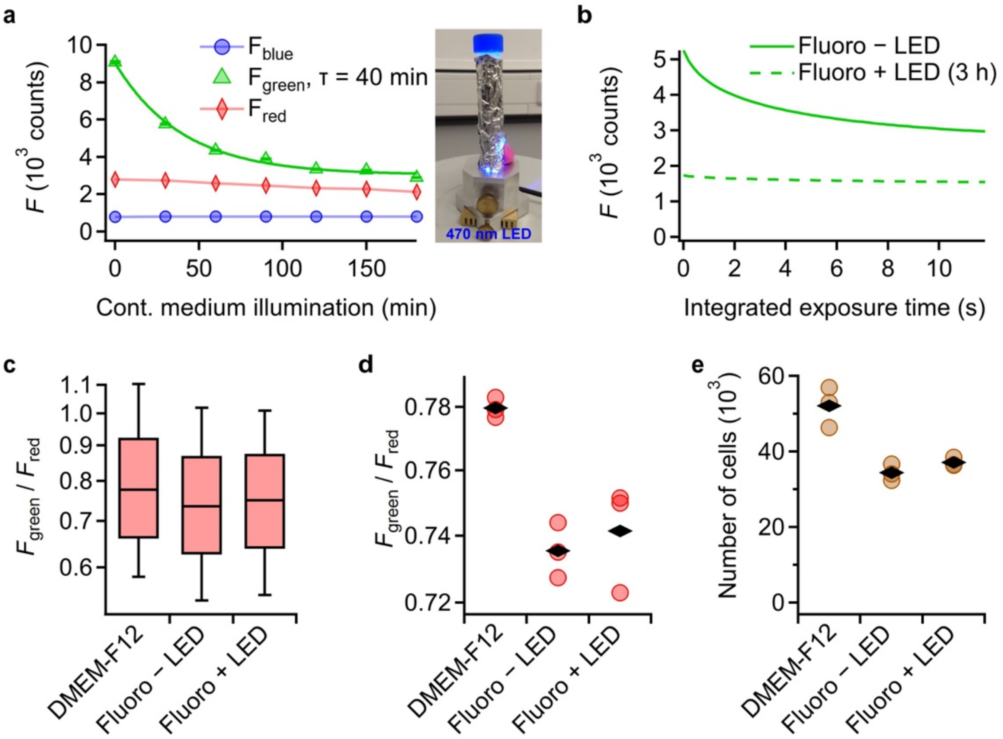

Supplementary Figure 9 | Bleached live-cell imaging medium does not compromise HEK293T cell viability. (**a**) Blue, green and red autofluorescence from images of FluoroBrite-DMEM + 10 % FCS (designated for live-cell imaging) as a function of the time for which the medium was illuminated (470 nm LED, 625 mW, as depicted on the right) before imaging with the same optical configuration used for high-content V_m imaging (**Fig. 2**) but at ambient temperature. Data are means ± sem of images at 4 different locations in the same cell-culture dish. The time course of F_{green} was fit with a single exponential; straight lines connect data points of F_{blue} and F_{red} for clarity. Photograph: sterile medium in a sealed 15-ml tube, wrapped in aluminum foil, was illuminated from the bottom with a 470-nm LED. (**b**) Transient of F_{green} of FluoroBrite DMEM as a function of integrated exposure time with the same optical configuration used in long-term live-cell experiments of fresh and pre-illuminated (470 nm for 3 h) FluoroBrite DMEM (as in **a**). (**c**) Boxplots of F_{green}/F_{red} from high-content V_m imaging of HEK293T cells transfected with a rASAP expression vector, treated and imaged according to **Fig. 2a**. 24 h before measurements, the cell-culture medium (DMEM-F12) was exchanged for DMEM-F12, FluoroBrite DMEM (Fluoro – LED), or bleached FluoroBrite DMEM (Fluoro + LED). (**d**) Median F_{green}/F_{red} values of 3 independent cell-culture dishes for each condition (as in **c**); cells > 4300 each. Black rhombi indicate mean values. (**e**) Number of cells (transfected and non-transfected) in a growth area of about 22 mm^2 after 24-h incubation in the depicted cell-culture medium. Black rhombi indicate mean values.

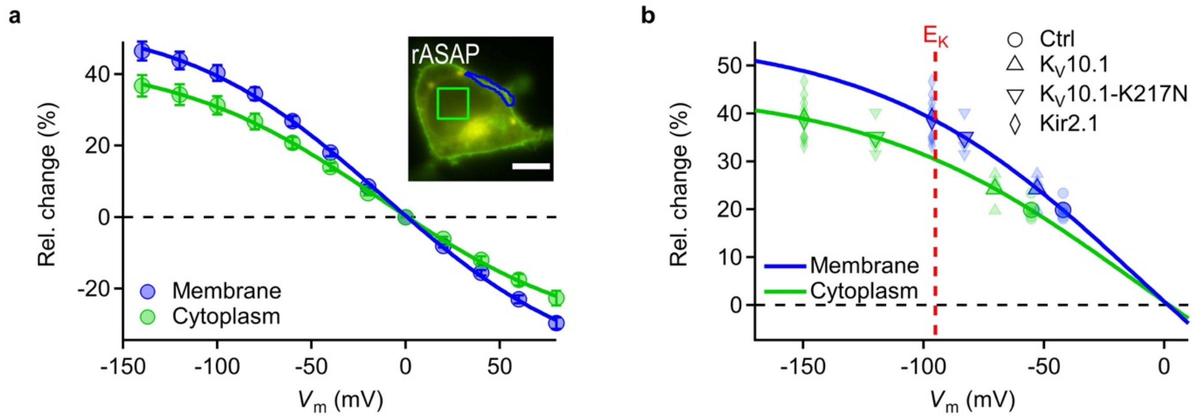

Supplementary Figure 10 | Impact of ROI selection on $F_{green}/F_{red}(V_m)$. (**a**) Percentage of F_{green}/F_{red} change relative to 0 mV as a function of V_m for HEK293T cells expressing rASAP with ROIs drawn at the locations indicated in the inset. Data sets were identical to data from **Fig. 1f** and are presented as means ± sem (n = 10) with superimposed Boltzmann-type functions (Eq. 1). Inset: Composite (green and red) images of a HEK293T cell under patch-clamp V_m control, expressing rASAP. ROIs for fluorescence signal extraction were drawn at the horizontal cross section at the edge of the cell (here termed "Membrane", *blue*) or in the center of the cross section (here termed "Cytoplasm", *green*). Scale bar, 10 µm. (**b**) Calibration curves from **a** with calculated V_m of cells expressing various K^+ channels. V_m of HEK293T cells was calculated by calibration to 0 mV with gramicidin. All other V_m were calculated from the relative change to HEK293T cells (Ctrl) assuming a constant V_m for HEK293T cells. The dotted red line shows the theoretical minimum V_m based on the estimated K^+ equilibrium potential (E_K) at 37 °C with 4 mM K^+ externally and 140 mM internally. Parameters of Boltzmann fits were R_{max} = 0.56, Δ_r = -178.6%, V_{half} = -11.5 mV, k_s = 54.0 mV for membrane localization and R_{max} = 0.79, Δ_r = -175.6%, V_{half} = -13.5 mV, k_s = 55.1 mV for cytoplasm. Dark symbols are the mean values, light symbols mark individual experiments.

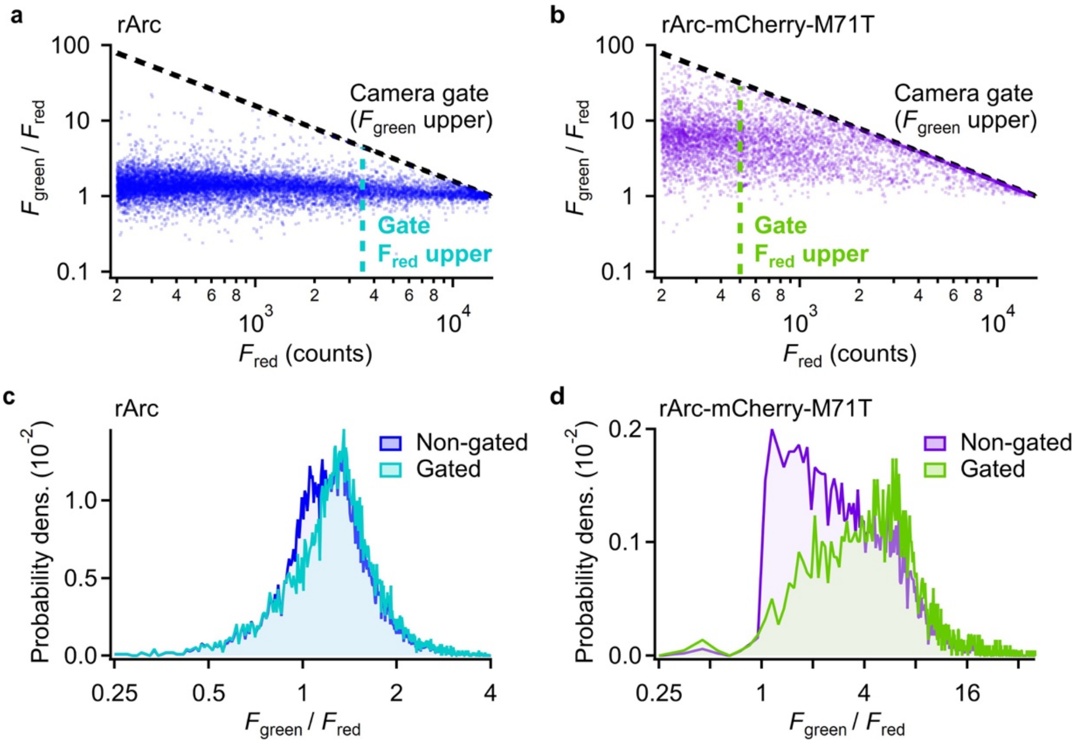

Supplementary Figure 11 | Correction for the limited camera dynamic range. (**a**, **b**) F_{green}/F_{red} as a function of F_{red} from images of HEK293T cells expressing rArc or rArc-mCherry-M71T. The saturation of individual camera pixels sets an upper limit to the measurable fluorescence (14 bit, 16,383 counts). Therefore, F_{green}/F_{red} cannot exceed a theoretical maximum as a function of F_{red}, here shown as thick black dashed line. Combinations of green and red fluorescent proteins yielding a large F_{green}/F_{red} value, in this case exemplified by rArc-mCherry-M71T, are most strongly affected by this technical cut-off. One way of limiting this bias is to eliminate data points with high F_{red} values. The vertical dashed cyan and green lines are such limits applied to rArc and rArc-mCherry-M71T data, respectively. Each data point represents one individual cell; cells > 5000 for both variants. Lower F_{red} gate was 200 counts in both cases; cells below this level were discarded. (**c**) Probability density of F_{green}/F_{red} of cells expressing rArc, considering all data points from **a** (non-gated) or only data below the manually set upper gate in **a** (3500 counts, cyan dashed line). (**d**) As in **c** but with rArc-mCherry -M71T; manual upper gate was 500 counts (green dashed line in **b**).

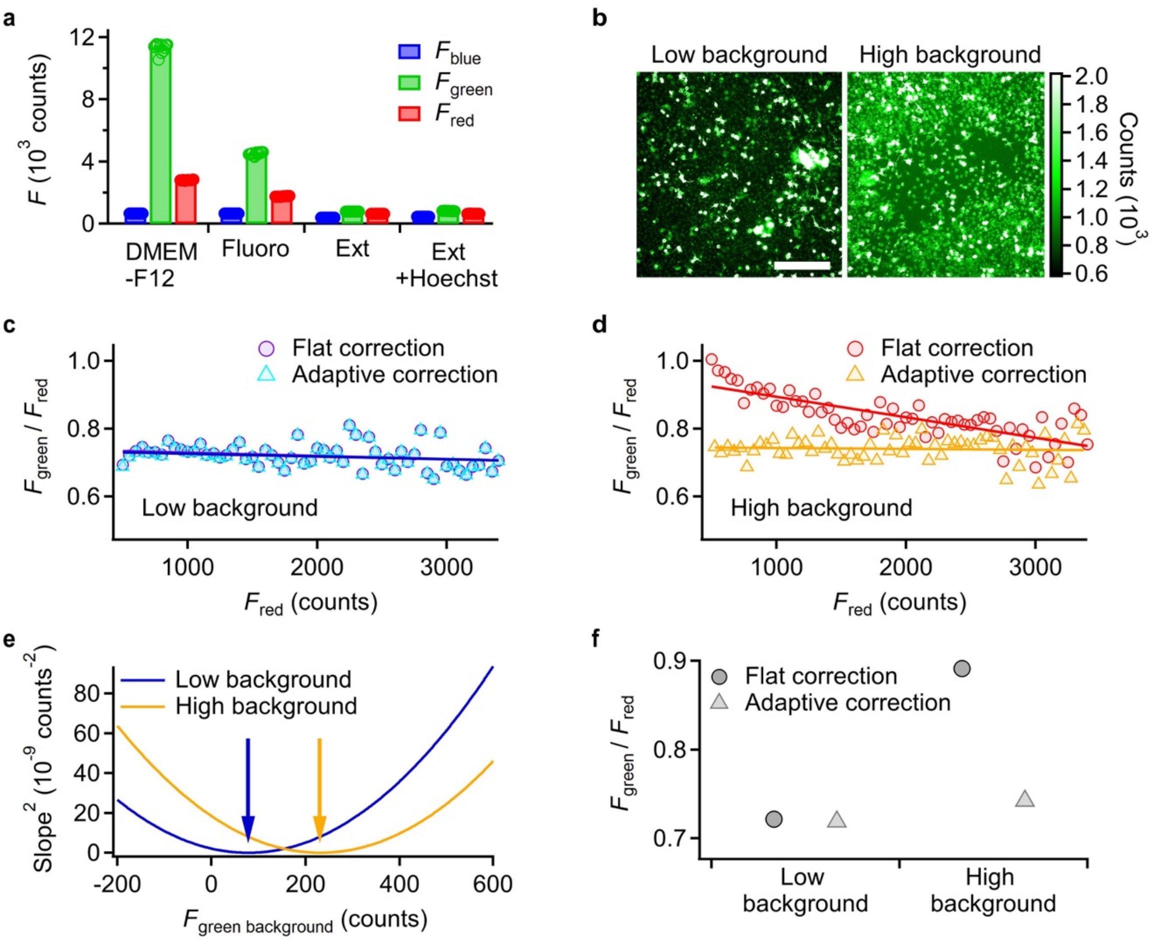

Supplementary Figure 12 | Correction of background subtraction. (**a**) Autofluorescence intensities of various cell-culture media in the blue, green and red channel, acquired with the same illumination protocol used for other live-cell imaging experiments at 37 °C. Data are average fluorescence values of images from 15-16 locations in a single culture dish containing 2 ml DMEM-F12, FluoroBrite DMEM, extracellular solution, or extracellular solution supplemented with 10 µg/ml Hoechst 33342. Data are means ± sem; circles mark individual experiments. (**b**) Representative F_{green} raw images of HEK293T cells expressing rASAP from two separate 35-mm culture dishes with different fluorescence background; calibration bar shows F_{green}. Scale bar, 100 µm. Background was partially subtracted using a rolling-ball algorithm on green and red images before data extraction. Cell medium (DMEM-F12) was exchanged for Ext + Hoechst 33342 30 min before data acquisition. (**c**) Median F_{green}/F_{red} as a function of F_{red} with flat background subtraction (75 counts) or using the location of the minimum from the curve in **e** as background (adaptive); linear fits are superimposed. (**d**) As in **c** but for a sample with larger background fluorescence; values are medians of the ratio binned according to the red intensity (bin width 50 counts). (**e**) Slope^2 as a function of subtracted background from a linear fit of F_{green}/F_{red} as a function of F_{red} (as in **c** and **d**) to determine the minima (arrows). (**f**) Median F_{green}/F_{red} values of samples with high or low background using flat or adaptive background subtraction; cells > 3600 each.
