# Supplementary material for: Monitoring of compound resting membrane potentials of cell cultures with ratiometric genetically encoded voltage indicators
Source: Commun Biol. 2021 Oct 7;4:1164. doi: 10.1038/s42003-021-02675-0 (PMC8497494; doi:10.1038/s42003-021-02675-0)
Supplement: Supplementary file 2 — Description of Additional Supplementary Files [file 42003_2021_2675_MOESM2_ESM.pdf]

Description of Additional Supplementary Files

File name: Supplementary Data 1

Description: Contains all data used to generate the graphs.

File name: Supplementary Data 2

Description: Numerical data values of all figures.

File name: Supplementary Data 3

Description: Sequences of DNA plasmids used in this study.
